# Strain Degeneration in *Pleurotus ostreatus*: A Genotype Dependent Oxidative Stress Process Which Triggers Oxidative Stress, Cellular Detoxifying and Cell Wall Reshaping Genes

**DOI:** 10.3390/jof7100862

**Published:** 2021-10-14

**Authors:** Gumer Pérez, Federico Lopez-Moya, Emilia Chuina, María Ibañez-Vea, Edurne Garde, Luis V. López-Llorca, Antonio G. Pisabarro, Lucía Ramírez

**Affiliations:** 1 Genetics, Genomics and Microbiology Research Group, Institute for Multidisciplinary Research in Applied Biology (IMAB), Public University of Navarre (UPNA), 31006 Pamplona, Spain; gumer.perez@unavarra.es (G.P.); chuina.110066@e.unavarra.es (E.C.); maria.ibanez@unavarra.es (M.I.-V.); edurne.garde@unavarra.es (E.G.); gpisabarro@unavarra.es (A.G.P.); 2 Laboratory of Plant Pathology, Department of Marine Sciences and Applied Biology, University of Alicante, 03690 Alicante, Spain; federico.lopez@ua.es (F.L.-M.); lv.lopez@ua.es (L.V.L.-L.)

**Keywords:** edible mushroom, ROS production, alternative oxidase, chitosan, growth rate

## Abstract

Strain degeneration has been defined as a decrease or loss in the yield of important commercial traits resulting from subsequent culture, which ultimately leads to Reactive Oxygen Species (ROS) production. *Pleurotus ostreatus* is a lignin-producing nematophagous edible mushroom. Mycelia for mushroom production are usually maintained in subsequent culture in solid media and frequently show symptoms of strain degeneration. The dikaryotic strain *P. ostreatus* (DkN001) has been used in our lab as a model organism for different purposes. Hence, different tools have been developed to uncover genetic and molecular aspects of this fungus. In this work, strain degeneration was studied in a full-sib monokaryotic progeny of the DkN001 strain with fast (F) and slow (S) growth rates by using different experimental approaches (light microscopy, malondialdehyde levels, whole-genome transcriptome analysis, and chitosan effect on monokaryotic mycelia). The results obtained showed that: (i) strain degeneration in *P. ostreatus* is linked to oxidative stress, (ii) the oxidative stress response in monokaryons is genotype dependent, (iii) stress and detoxifying genes are highly expressed in S monokaryons with symptoms of strain degeneration, (iv) chitosan addition to F and S monokaryons uncovered the constitutive expression of both oxidative stress and cellular detoxifying genes in S monokaryon strains which suggest their adaptation to oxidative stress, and (v) the overexpression of the cell wall genes, *Uap1* and *Cda1*, in S monokaryons with strain degeneration phenotype indicates cell wall reshaping and the activation of High Osmolarity Glycerol (HOG) and Cell Wall Integrity (CWI) pathways. These results could constitute a hallmark for mushroom producers to distinguish strain degeneration in commercial mushrooms.

## 1. Introduction

Filamentous fungi frequently degenerate during their maintenance in culture by showing loss of the ability to reproduce sexually or asexually [1]. Degeneration of fungal cultures has been related to a cellular accumulation of Reactive Oxygen Species (ROS). In *Aspergillus nidulans* mycelia with fluffy sectors, cytochrome c release, calcium overload, and upregulation of apoptotic genes revealed a link between oxidative stress and fungal culture degeneration [2]. Moreover, in *Metarhizium anisopliae*, sectorial cultures showed syndromes associated with aging, upregulation of genes involved in deoxidation and self-protection (i.e., Heat-Shock Proteins, HSPs), physiological adaptations such as cell structure reorganization, and the activation of different signaling pathways. [3]. In *Volvariella volvacea*, an edible basidiomycete, prolonged subculturing of mycelia for six generations led to a decrease of growth rate, mycelia biomass, protein, carbohydrate, polyphenol, flavone, and total amino acid and mineral element content. The decrease of these variables indicates an oxidative stress process characteristic of strain degeneration [4].

Oxidative stress was defined as a disturbance in the balance between ROS production and antioxidant defenses [5]. ROS are produced by cellular metabolic processes and environmental factors and encompass free radicals, including the superoxide anion (O_2_^−^) and hydroxyl radical (^•^OH), as well as nonradical but highly reactive molecules formed upon incomplete reduction of oxygen, such as hydrogen peroxide [6]. ROS, have been viewed as harmful due to their stronger reactivity with molecular oxygen, their capacity to damage cellular components, and their ability to disturb cellular homeostasis leading to cell aging or death.

Low ROS levels are necessary for critical biological processes such as cell proliferation or differentiation [7,8] because they act as signaling molecules to induce many physiological processes [9,10] and ensure cellular redox homeostasis, avoiding disorders that influence cell growth or aging. High levels of ROS are toxic and trigger different signaling pathways. Examples are the retrograde response pathway [9,10,11,12], the Unfolded Protein Response (UPR) [13,14,15], ER stress-activated [16,17], TOR [18,19], and different MAP kinase pathways [20,21,22]. These force the cell to reach a dynamic equilibrium between ROS generation and elimination that is accomplished by detoxifying enzymes, such as superoxide dismutase, catalase, glutathione peroxidase, components of glutathione/glutaredoxin and thioredoxin [23]. In fungi, oxidative stress has been studied by analyzing the effect of free radicals, such as O_2_^−^ and ^•^OH, the highly reactive H_2_O_2_, salts [24], heavy metals [25], temperature [26], and fungicides such as fluconazole [27] and chitosan [28], among others, on the upregulation of genes to alleviate it.

Chitosan is a natural polycationic linear polymer of β-1,4 glucosamine subunits, and it is a partly deacetylated form of chitin [29]. It was considered a biocidal [30,31,32,33,34], able to elicit plant defense mechanisms [35,36,37] and damage the plasma membranes of both bacteria [32] and yeast [38]. Its biocompatibility, nontoxicity, low allergenicity, and biodegradability have permitted its application in different uses [39]. Different chitosan sensitivity was observed in fungi belonging to different classes (*Sordariomycetes*, *Oomycetes,* and *Basidiomycetes*). In this sense, chitosan resistance was observed in the *Cordycipitaceae* and the *Clavicipitaceae* families, to which the entomopathogenic (*Beauveria bassiana* and *Lecanicillium psalliotae*) and the nematophagous egg-parasitic (*Pochonia* sp. and *Paecilomyces lilacinus*) fungi belong [40].

*Pleurotus ostreatus* is a filamentous, nematophagous, lignin degrader, and edible basidiomycete with important biotechnological properties [41,42,43]. As a tetrapolar basidiomycete, the dikaryotic mycelia results from the fusion of two compatible monokaryons, derived from the mitosis of haploid spores formed in basidia after nuclei fusion to form a unique diploid nucleus, which immediately undergoes meiosis [44].

The *P. ostreatus* strains used for mushroom production are maintained in continuous culture on different solid culture media, and it is frequently observed that mycelia display signs of strain degeneration. For longer than 30 years, the dikaryotic strain *P. ostreatus* DkN001 has been used as a model organism in our lab for different purposes.

In this sense, we have developed different genetic and molecular tools [45,46,47,48,49] that have allowed the construction of the first genetic linkage map of *P. ostreatus*. This map was used to place orderly qualitative and quantitative traits. Examples are mating type [45], hydrophobins [46], laccases [46], manganese peroxidases [46], and copper transporter [50] genes, telomeric and subtelomeric sequences [51], and QTLs such as monokaryotic and dikaryotic growth rate, mushroom yield production, earliness, fruiting body number, color and fleshiness [52,53].

The dedikaryotization of the strain dkN001 yielded two monokaryotic strains (protoclones): the fast-growing monokaryon MkPC9 and the slow-growing MkPC15, identified by molecular markers [54]. The growth rate quantitative traits were inherited in the progeny and were mapped to different chromosomes. The growth rate allowed the clustering of the progeny into two groups: fast- (F) and slow-growing (S) monokaryons [52]. Genome sequencing of both protoclones (Joint Genome Institute, JGI) revealed a high degree of synteny between them. A whole-genome transcriptome analysis profile revealed 11,820 genes and a substantial population of transposable elements. The sequence of both protoclones also made possible the identification of Single Nucleotide Polymorphisms SNPs [55], which permitted identifying the parental origin allele in monokaryons derived by meiosis from dkN001. This information enabled us to pose questions and design experiments to uncover the underlying genomic differences between fast- and slow-growing monokaryon strains, which showed strain degeneration.

For that purpose, in this work, we analyzed two subpopulations of eighteen full-sib monokaryons obtained from the dikaryotic strain DkN001 in 2016, as well as their monokaryotic protoclones MkPC9 and MkPC15. The progeny was clustered according to their growth rates as F- and S-subpopulations. Growth rates, biomass production, and light microscope studies were performed. A correlation between monokaryon genotype versus phenotype was carried out in both subpopulations to determine whether any relationship existed to warrant their performance. Finally, whole-genome transcriptome analyses of the full-sib monokaryons and their parental protoclones were carried out. The constitutive expression of two genes, *Uap1* (a UDP-N-acetylglucosamine diphosphorylase, EC 2.7.7.83) and *Cda1* (a chitin deacetylase, EC 3.5.1.41), both involved in cell wall reshaping in slow-growing monokaryons, moved us to study the effect of chitosan, a biocidal compound with important medicine and agriculture properties [56], which drastically reduced the growth rate in these monokaryons.

## 2. Materials and Methods

### 2.1. Fungal Strains and Growth Conditions

The *P. ostreatus* dikaryotic strain DkN001 (Spanish Type Culture Collection accession CECT20600), and the monokaryotic protoclones MkPC9 (CECT20311, fast-growing) and MkPC15 (CECT20312, slow-growing) obtained by dedikaryotization of the strain DkN001 were used in this work [54]. The mating of MkPC9 and MkPC15 restores the dikaryotic strain DkN001. A progeny of 60 monokaryons derived from single spores obtained after meiosis of the strain DkN001, was isolated in 2016. All monokaryons were cultured on Malt Extract Solid Medium (MESM: malt extract, 20 g/L; and bacteriologic agar, 15 g/L). Linear growth measurements were performed until mycelia thoroughly colonized the 9 mm diameter Petri dishes. The growth rate value of each strain is the average of three replicates. Samples for biomass production, RNA-seq, and RT-qPCR experiments were obtained from the protoclones, MkPC9 and MkPC15, and monokaryons of the Fast (F) (Mk01, Mk02, Mk06, Mk08, Mk13, Mk20, Mk24, Mk27, Mk28) and Slow (S) (Mk61, Mk62, Mk69, Mk74, Mk75, Mk76, Mk77, Mk82, Mk83) subpopulations. Three replicates of each strain were grown for six days in Erlenmeyer flasks containing 100 mL of SMY medium (1% Sacarose, 1% Malt Extract, and 0.4% Yeast Extract), and incubated in the dark at 24 °C under orbital shaking (125 rpm) to allow growth adaptation to the culture medium. The adapted cultures were homogenized, and a 15 mL inoculum was used to inoculate flasks containing 135 mL of liquid SMY medium and this was incubated in static Submerged Fermentation (SmF) in darkness for seven days at 24 °C. Mycelia were collected, weighed, frozen in liquid nitrogen, and stored at −80 °C for later use. The correlation between the linear growth rate of the monokaryons on Petri dishes and biomass production was evaluated using the Spearman correlation statistic ρ. It was assumed that both variables were correlated when ρ was >0.65 and the *p*-value was <0.05.

### 2.2. RNA Extraction, Library Construction, and Sequencing

For RNA analysis, three biological replicates of each strain were grown in SMY as described above. Each replicate was split into two halves: one of them was stored at −80 °C for RT-qPCR experiments, and the second half of replicates were pooled, ground in a sterile mortar using liquid nitrogen, and stored at −80 °C to be used for transcriptome analysis. A fungal RNA E.Z.N.A. kit (Omega Bio-Tek, Norcross, GA, USA) was used to obtain the total RNA, the quality of which was determined by electrophoresis on 1% (w/v) agarose gels. Technical RNA duplicates were performed to assess RNA concentration measured by Qubit^®^ 2.0 fluorometer using a Qubit^®^ RNA Assay kit (Invitrogen, Life Technologies Corporation, USA) and Bioanalyzer (version 2100). All samples had 260/280 ratios greater than 2.0 and RNA integrity numbers (RIN) ≥ 8.8. All mRNA libraries were constructed using the TruSeq^®^ RNA sample (Illumina^®^, Inc., San Diego, CA, USA), following the manufacturer’s instructions. The sequencing was performed with an Illumina HiSeq 2000 system using 75 bp paired-end reads.

### 2.3. Analysis of Sequencing Data

Data analysis was performed according to Castanera et al. [55]. Briefly, mRNA-seq data were filtered for quality using FastQC and trimmed with Trimmomatic 5. The resulting reads from all libraries were mapped to the *P. ostreatus* reference genomes (PC15 v2.0, www.genome.jgi.doe.gov/PleosPC15_2/PleosPC15_2.home.html, accessed on 3 October 2018, and PC9 v1.0 https://genome.jgi.doe.gov/PleosPC9_1/PleosPC9_1.info.html: MycoCosm portal, accessed on 3 October 2018 [57]) using STAR v2.3.1 [58] restricted to single hit mapping. Expression levels were quantified using a Python script to calculate RPKMs (www.sandberg.cmb.ki.se/media/data/rnaseq/rpkmforgenes.py, accessed on 5 October 2018). Gene identification numbers (IDs) correspond to the reference genome (PC15 v2.0, www.genome.jgi.doe.gov/PleosPC15_2/PleosPC15_2.home.html, accessed on 11 October 2018).

### 2.4. SNPs and Crossover Detection

Single Nucleotide Polymorphisms (SNPs) between protoclones MkPC9 and MkPC15 were detected using RNA-seq data. Alleles of MkPC9 and MkPC15 were identified by reciprocal BLAST (Basic Local Alignment Search Tool, [59]). Each generated alignment was analyzed to identify genomic polymorphisms between the two protoclones and saved in a VCF file. RNA-seq data of the 18 monokaryons of this study were mapped to the reference genome (MkPC15) using the STAR program [58]. Each mapping was done with the VCF file using the GATK HaplotypeCaller program [60,61] to identify each SNP and determine the allele origin.

### 2.5. Correlation Analysis between Growth Rate and Gene Expression (RPKMs)

The correlation between gene expression (RPKMs) and growth rate (mm/day) was studied using the Spearman correlation statistic, ρ. Values of ρ greater than 0.65 indicate a positive correlation (PC) between a specific gene and growth rate, whereas values lower than −0.65 indicate a negative correlation (NC). The False Discovery Rate (FDR method, [62]) cut off used was <0.05 and the *p*-value was <0.005. To identify the genes differentially expressed (DEGs) between F- and S-subpopulations, the abundance of the transcripts was calculated according to the Reads per Kilobase of exon per Million mapped reads (RPKMs) method. R statistical package software EdgeR (Empirical Analysis of Digital Gene Expression in R, http://www.bioconductor.org/packages/2.12/bioc/html/edgeR.html, accessed on 11 October 2018) was used for differential expression analysis [63]. Only genes with a Fold Change (FC) of >1.5, a FDR cut off of <0.05 and *p*-value of <0.005 were used for the analyses.

### 2.6. Functional Analysis of Genes

Correlated and DEG genes were functionally annotated using OmicsBox software [64], and their Eukaryotic Orthologous Group (KOG) classification was performed using EggNOG-mapper (OmicsBox-Biobam Bioinformatics). Genes were blasted against the NCBI non-redundant protein database, and the InterProScan was used to search domains. Allocation by GO domains into the different categories: Biological Process (BP), Cellular component (CC), and Molecular Function (MF), are shown according to generic terms in the OmicsBox software.

### 2.7. RT-qPCR Analysis

The relative expression level of selected genes was analyzed to validate RNA-seq data via quantitative reverse transcription PCR (RT-qPCR). Total RNA (800 ng per sample) was reverse transcribed into cDNA using an iScript cDNA synthesis kit (Bio-Rad, Laboratories, Inc. Hercules, CA, USA). Reverse transcription (RT) was carried out in a thermal cycler (MJ Research, Inc. Waltham, MA, USA) using the following program: 5 min at 25 °C, 30 min at 42 °C, and 5 min at 85 °C. RT-qPCRs were performed in a CFX96 real-time system (Bio-Rad, Laboratories, Inc.) using SYBR green dye to detect product amplification. Each reaction mixture contained 10 μL iQ SYBR green supermix (Bio-Rad, Laboratories, Inc.), 2 μL of 3 μM forward and reverse primers, 1 μL of a 1:20 dilution of the RT product, and 5 μL of sterile water. The amplification program consisted of 5 min at 95 °C and 40 cycles of 15 s at 95 °C, 30 s at 63 °C, and 15 s at 72 °C, followed by a final melting curve analysis in which the temperature was increased in increments of 0.5 °C every 5 s in a linear gradient from 65 to 95 °C. Three biological replicates were used for each monokaryon. Relative gene expression was determined by the 2^−∆∆Ct^ method using the GenEx software for processing and analysis of qPCR data [65]. All Primer pairs used in this work are shown in Table S1, and their amplification efficiencies were greater than 95%. qPCR data were normalized using three reference genes (Table S1) according to Castanera et al. [66].

### 2.8. ROS Detection Assay

Monokaryons were grown on MESM Petri dishes until they reached a diameter of 30 ± 3 mm. For superoxide radical (O2^−^) detection, the colonies were flooded with 10 mL of staining solution containing 5 mM 3-(N-morpholino) propane sulfonate NaOH buffer, pH 7.6, and 2.5 mM of Nitro Blue Tetrazolium (NBT, Sigma-Aldrich, St. Louis, MO, USA) and incubated at 24 °C for 30 min. The staining solution was discarded, and plates were incubated for 1.5 h at 24 °C in the dark.

For H2O2 detection, a similar procedure to the NBT was applied using a staining solution of 100 mM potassium phosphate buffer, pH 6.9, 2.5 mM Diaminobenzidine tetrachloride (DAB, Sigma-Aldrich), and 5 purpurogallin units/mL of horseradish peroxidase (Type VI, Sigma-Aldrich) freshly prepared and shielded from light to avoid spontaneous photo-oxidation. The reaction produced a brownish polymer in the mycelium.

### 2.9. Lipid Peroxidation Assay

Lipid peroxidation was measured in all the monokaryons of the F- and S-subpopulations by the thiobarbituric acid (TBA) test, according to Hodges et al. [67]. Mycelia were handled as previously described for RNA-seq experiments, frozen and ground in a sterile mortar in the presence of liquid nitrogen, and homogenized in 1 mL prechilled 0.1% (w/v) trichloroacetic acid (TCA) solution. Homogenates were centrifuged at 20,000× *g* for 5 min at 4 °C, and 750 μL of the supernatant were mixed with 750 μL of the Reagent Solution II (RSII: RSI (20% w/v TCA and 0.01% v/v Butylated hydroxytoluene (BHT)) + 0.65% w/v TBA), mixed vigorously and incubated at 95 °C for 25 min. The reaction was stopped by placing the tubes in ice. Samples were centrifuged as above, and the supernatants were transferred to new tubes. Absorbances were read with a Shimadzu spectrophotometer Uvi-1900 (Shimadzu Corporation, Kyoto, Japan) at 440 nm (sugar absorbance), 532 nm (maximum absorbance of pinkish-red chromagen, the product of the reaction of malondialdehyde (MDA) with TBA) and 600 nm (turbidity). The reference solution consisted of 0.1% (w/v) TCA. MDA equivalents were estimated as follows:

MDA equivalents (nmol/mL) = [(A − B)/157000] × 106 where A = [(Abs 532RSII − Abs 600RSII)] and B = [(Abs 440RSII − Abs 600RSII) × 0.0571]

MDA equivalents (nmol/g Fresh Weight) = MDA equivalents (nmol/mL) × total volume of the extracts (mL/g) FW.

### 2.10. Chitosan Treatments

Sensitivity to chitosan was analyzed in monokaryons of F- and S-subpopulations grown on Petri dishes containing MESM amended with 0.5, 1, and 2 mg/mL chitosan concentrations. Medium molecular weight chitosan (70 kDa) with an 82.5% deacetylation degree (T8s; Marine BioProducts GmbH; Bremerhaven, Germany) was used. Chitosan was prepared according to Palma-Guerrero et al. [34]. The chitosan effect was evaluated by measuring the reduction in the mycelium growth rate of each strain grown for 10 days in Petri dishes containing different chitosan concentrations. Three replicates of each monokaryon were evaluated. Data obtained were analyzed with IBM SPSS statistics 27.0 software (IBM Corp. Released 2020. IBM SPSS Statistics, Version 27.0. Armonk, New York: IBM Corp) using the Scheffe post hoc statistic to compare all possible simple and complex pairs of means between the different doses and control of each monokaryon. Mycelia were harvested to be used in RT-qPCR experiments as described above.

### 2.11. Protein Purification and Immunoblot Analysis

Phosphorylation of Mitogen-activated Protein Kinases (MAPKs) was analyzed in monokaryons of F- and S-subpopulations. Mycelia were obtained as described previously for RNA-seq experiments. Briefly, frozen mycelia maintained at −80 °C were ground in a mortar, and resuspended in protein extraction buffer containing 10 mM HEPES, 50 mM KCl, 1 mM EGTA, 1 mM MgCl_2_, Protease Inhibitor Cocktail tablets (Roche Diagnostics, SL, Basel, Switzerland) and PhosSTOP Phosphatase Inhibitor Cocktail tablets (Roche Diagnostics, SL). Samples were centrifuged to pellet debris, and the protein concentration of the soluble fraction was measured by Bradford assay (Bio-Rad Laboratories, Inc.). Twenty-five micrograms of total protein were separated in a 10% SDS-PAGE gel and transferred to nitrocellulose membranes for Western blot analysis using a Trans-Blot Turbo Mini Nitrocellulose Transfer Pack following the manufacturer’s instructions (Bio-Rad Laboratories, Inc.). Phosphorylation of TEY and TGY motifs of MAPKs were detected with phosphor-p44/42 (ERK1/2 homologous) and -p38 (Hog1 homologous) MAP kinase antibody kits (Cell Signalling Technology, Danvers, MA, USA) following the manufacturer’s instructions. An α-tubulin monoclonal antibody (Sigma Aldrich) was used for the loading control.

## 3. Results

### 3.1. Growth Rate as a Feature to Identify Monokaryons

**Figure 1:**
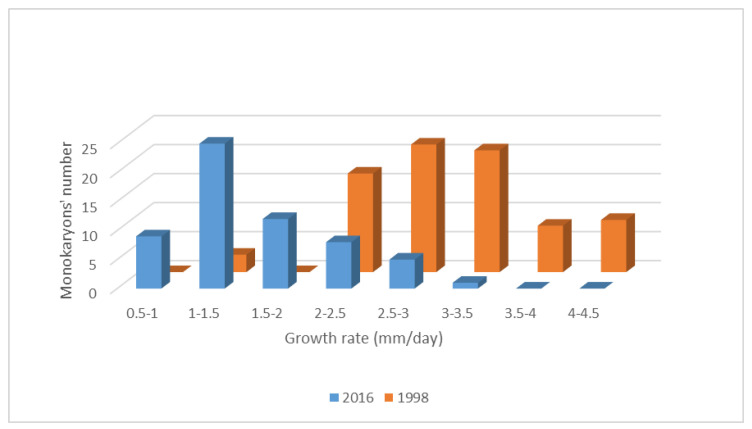
Comparison of the growth rates of two monokaryotic progenies obtained from DkN001 in 1998 (80 monokaryons, orange) and 2016 (60 monokaryons, blue). Growth rate data were grouped in intervals and measured in mm/day as described in Materials and Methods.

We studied the growth rates on malt extract solid medium (MESM) of a progeny of 60 monokaryons obtained in 2016 from the dikaryotic strain *P. ostreatus* DkN001. The growth rate of each monokaryon (Mk) was the average of three biological replicas. The growth rates ranged from 0.56 to 3.08 mm/day (Figure S1) and were highly different from those of the monokaryotic progeny derived by meiosis from the same DkN001 in 1998 (1.02 mm/day and 4.2 mm/day, data not shown). The growth rate values of monokaryons of this new progeny showed no normal distribution (Figure 1), and the nonparametric Kruskal-Wallis test was used to group monokaryons according to their growth rates. As described previously, two subpopulations of nine monokaryons (F fast and S slow) were established (Table S2).

The hyphal width and the distance between septa in the mycelia of fast and slow monokaryons were analyzed. No significant diameter differences between fast and slow monokaryons were observed (5.51 ± 1.19 μm versus 5.1 ± 1.2 μm), whereas striking differences were found in the distance between septa in fast-growing (43.56 ± 5.25 μm) versus the slow-growing monokaryons (23.41 ± 2.01 μm) (Figure S2).

### 3.2. Fast-Growing Monokaryons Are Higher Biomass Producers

The phenotype differences observed between both subpopulations prompted us to design a whole-genome transcriptome analysis of monokaryons of both subpopulations to uncover their molecular bases. Because the cultures made on a solid media did not yield enough biomass for the transcriptome analysis, each monokaryon was cultured in SmF for seven days. The Spearman coefficient was used to assess the correlation between growth rate and biomass production, and the data obtained revealed a positive correlation value of ρ = 0.7 (*p*-value < 0.005) between both variables. Monokaryons of both subpopulations showed a different growth behavior: those of the F-subpopulation developed aerial mycelia while those of S-subpopulation showed partly floating or completely submerged mycelia.

### 3.3. RNA Sequence Analysis Allows Detection of SNPs and Crossover Locations and Reveals Differences between F- and S-Subpopulations

The whole transcriptome was analyzed in protoclones MkPC9 and MkPC15, nine fast- and nine slow-growing monokaryons of the progeny (F- and S-subpopulations).

**Figure 2:**
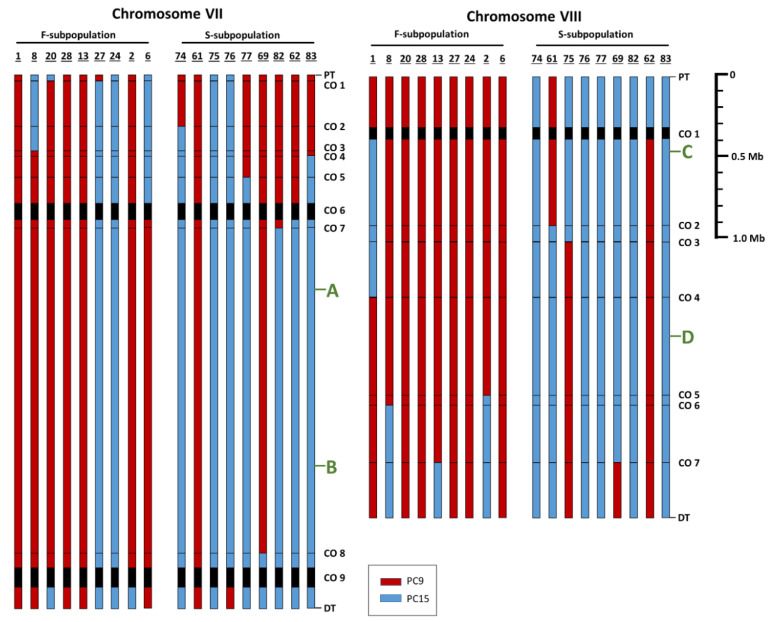
SNP haplotypes of chromosomes VII and VIII for the nine fast- and nine slow-growing monokaryons under study. For each monokaryon, the colors indicate the chromosome fragments derived from MkPC9 (red) and MkPC15 (blue) protoclones. CO indicates crossover regions (listed in sequential order) from the Proximal (PT) to the Distal Telomere (DT). Black lines and areas correspond to crossover regions smaller and greater than 50 kb, respectively. Green letters indicate the mapping position of *Sod1* (A), *Aox* (B), *Cyc7* (C), and *Cox5B* (D).

RNA-seq analysis uncovered SNPs (Single Nucleotide Polymorphisms) between MkPC9 and MkPC15, which were detected in the progeny (Figure 2). SNP data (about 190,000) allowed: (i) the identification of the parental origin of each allele, (ii) the construction of an array of SNPs in both subpopulations, and (iii) the location of crossover (CO) regions (Figure S3) in each chromosome of the *P. ostreatus* parental strain DkN001. Allele differences were observed in 7381 out of the 11,820 genes of the whole genome of *P. ostreatus* (transposons not considered).

Ninety-two crossovers were distributed along the 11 chromosomes of *P. ostreatus* DkN001. Of the genome regions spanning crossovers, 28% (26/92 crossovers) were enriched with transposable elements (Table S3) such as gypsy retroelements and LTR-retrotransposons. No correlation was observed between the crossover number and the chromosome length. Chromosomes V and XI are the fourth- and the sixth-longest chromosomes of *P. ostreatus* MkPC15 and showed the highest percentage of crossover (≈12%). The mating-type carrying chromosomes III and IX showed 9.78% of crossovers (Table S4). Other chromosomes with a similar crossover abundance were chromosomes VI and VII, which are highly polymorphic. A high frequency of crossovers was observed at the sub-telomeric regions of chromosomes I, II, VII, IX, and X (data no shown). SNPs analysis showed that Mk83 was achiasmatic in five of the 11 chromosomes (III, VI, VIII, IX, and XI), four of them were of the MkPC9 type.

### 3.4. Different Gene Expression of the F- and S-Subpopulations

Differentially Expressed Genes (DEGs) were analyzed in the F- and S-subpopulations to capture differences between monokaryons belonging to each category. The whole transcriptome analysis yielded 531 genes differentially expressed (*p*-value < 0.005) with a fold change of >1.5 in both subpopulations. More than 50% of the DEGs were placed on chromosomes I (12.6%), III (14.1%), VII (11.3%), and VIII (16.9%). Out of 531 DEGs, 204 (38%) were upregulated in the F-subpopulation (F-DEGs) (Table S5) and 327 (61.6%) DEGs in the S-subpopulation (S-DEGs) (Table S6).

The Spearman correlation coefficient was used to determine if any correlation existed between gene expression (RPKMs) and monokaryon growth rates (mm/day). There were 900 genes that correlated with the growth rate in monokaryons of both subpopulations; 808 of them were Positively Correlated (PC genes, ρ > 0.65, Table S7), and 92 were Negatively Correlated (NC genes, ρ < −0.65, Table S8) with the growth rate, both with a *p*-value < 0.005.

**Figure 3:**
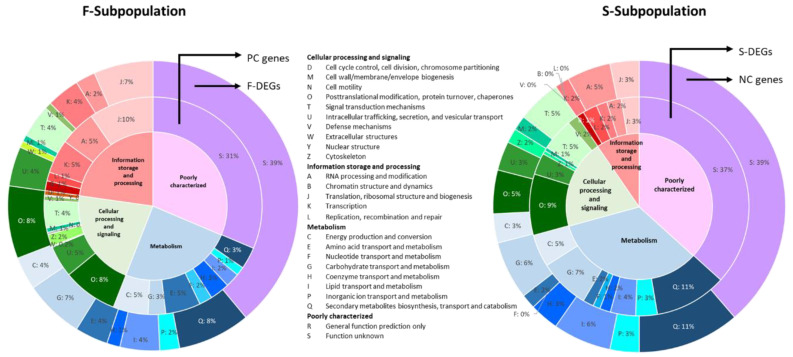
Eukaryotic Orthologous Group (KOG) classification representing 25 KOG functional categories. The numbers in the picture indicate the percentage of genes assigned to each KOG category in this study. The order of the inner and outer circles in both graphs were made based on the best fit with the central circle.

KOG (Eukaryotic Orthologous Group) analysis was used to determine the categories to which DEGs and PC and NC genes belong. The results obtained showed, in the F-subpopulation, 615/808 PC genes (76%) and 136/204 F-DEGs (66%) assigned to KOG classifications (Figure 3, Table S9). The most representative KOG categories were translational, ribosomal structure and biogenesis (KOG:J), post-translational modification, protein turnover, chaperones (KOG:O), and function unknown (KOG:S) (Figure 3, Table S9). In these categories genes such as translation elongation factors (EF-Tu, ID:1092267 and EF-Ts, ID:49695), several ribosomal proteins (Img2, ID:1031833; RpL19, ID:1075979; RpL34, ID:1087865 and RpS8, ID:1050332), t-RNA synthetases (Dia4, ID:1101995; Ths1, ID:1032700), a t-RNA ligase (Vas1, ID:1094347), a tRNA methyltransferase (ID:1073433), several chaperones (Scj1, ID:1067605; Sba1, ID:1088350; Zuo1, ID:1110221), and Hsp70 (Kar2, ID:62237; Ssb1, ID:1052407; Ssz1, ID:1049195) and Hsp90 heat shock proteins (ID:1092760) were found. (Tables S5 and S7).

In the S-subpopulation, 62/92 NC genes (67%) and 188/327 S-DEGs (57%) were assigned into KOG classifications (Figure 3, Table S9). The most representative categories were carbohydrate transport and metabolism (KOG:G), secondary metabolites biosynthesis transport and catabolism (KOG:Q), lipid transport and metabolism (KOG:I), and the last corresponded to genes with unknown function (KOG:S). Genes belonging to these categories were an alternative oxidase (*Aox*, ID:1106868), ABC transporter genes (*Pdr15*, IDs 38832 and 1091717; *Yor1*, ID:1106912), genes related to detoxification and cell protection (*Trx1*, ID:1097745; *Sfa1*, ID:1063808), several cytochrome p450s (IDs:155371, 1114747, 1062448, 1047307), two genes of the multicopper oxidase family (*Lacc3*, ID:1102751; *Lacc4*, ID:1077328), an exo-beta-1,3 glucanase (Gh5, ID:1033087), putative transglycosidases (Gh16, IDs:1070273 and 1079606), putative channel-like proteins belonging to the MIP aquaporin family (IDs: 160783 and 1073531), transporters for riboflavin (*Mch5*, ID:1033403) and D-galactonate (*DgoT*, ID:1102187), genes related to fungal cell wall metabolism (such as chitin deacetylases, *Cda1*, IDs: 1067159 and 1078467 and UDP-N-acetylglucosamine diphosphorylase, *Uap1*, ID: 1107060), putative mitochondrial chaperones (*Bcs1*, IDs: 36199, 1065807 and 1105500), a transmembrane GTPase-mitofusin (*Fzo1*, ID:1058073), an autophagy aspartic protease (*Ap1*, ID:1033895), different genes involved in ergosterol biosynthesis (such as *Erg1*, ID:1046279; *Erg9*, ID:1084890 and *Erg11*, ID: 1061968), and a stearoyl ∆^9^ desaturase (*Ole1*, ID:1050437) (Tables S6 and S8).

Venn diagrams were constructed to untangle any overlapping between PC and NC genes with growth rate and DEGs in both subpopulations. There were 114 and 70 genes that overlapped between the categories that were compared (Figure S4). GO terms were assigned to these genes by using the OmicsBox software. Comparisons between overlapping DEGs in PC and NC genes (Tables S5 and S6, and Figure S5) showed that the most representative category in both populations was Cellular Component (CC), and, within it, the function of the Integral Component of Membrane with 20 and 17 genes in the F- and S-subpopulations, respectively, although the DEGs found in each subpopulation were not the same. When comparisons were performed in the category of Biological Process (BP), functions related to protein synthesis were most prominent in the F-subpopulation.

By contrast, the most relevant function in the BP and Molecular Function (MF) categories in the S-subpopulation were transmembrane transport (examples of genes in this function are: several pleiotropic drug resistance ABC transporters, *Pdr15* and MFS polyamine transporters, *Tpo1*), and metal ion binding (wherein genes such as an alternative oxidase *Aox*, a flavoprotein *Aif1*, ID:1113817, or a histone acetyltransferase coactivator of SAGA complex *Ada2*, ID:1112521, could be found).

#### 3.4.1. Validation of DEGs by RT-qPCR

**Figure 4:**
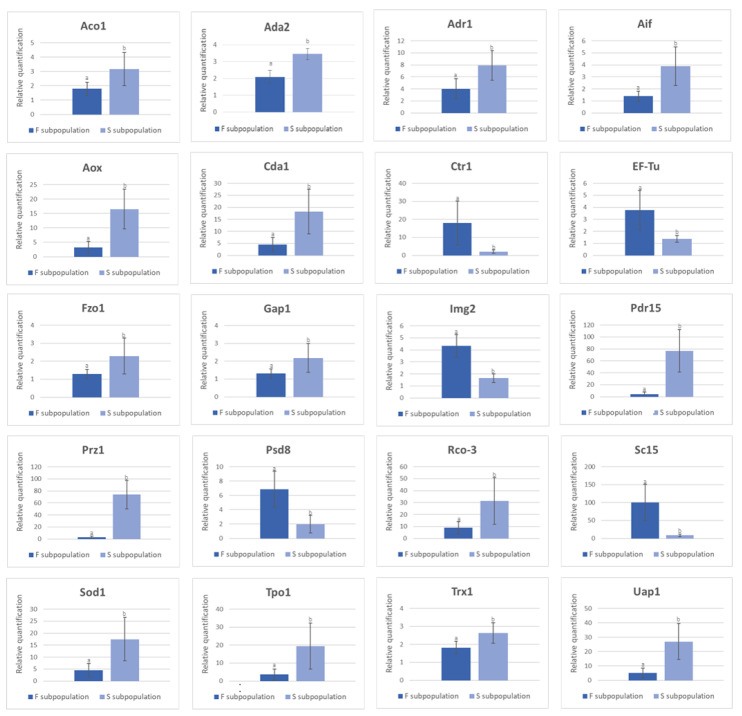
RT-qPCR validation of differentially expressed genes in monokaryons of F- and S-subpopulations. The results are reported as mean ± standard deviation (SD) of nine fast (F-subpopulation) and nine slow (S-subpopulation) growing monokaryons. *Actin1*, *Gaph1,* and *Sar1* were used as reference genes. Three biological replicates were used for each monokaryon. Genes are represented by their abbreviation, as indicated in Table S1. Lower-case letters indicate statistically significant expression differences between F- and S-subpopulations (Student *t*-test, *p*-value < 0.005).

In order to validate RNA-seq experiments, 20 DEGs belonging to different KOG categories in both subpopulations were studied. The relative expression data provided by RT-qPCR were consistent with their genome RNA transcriptome profile detected by RNA-seq and confirmed the upregulation of the analyzed genes (Figure 4).

The results showed a strong correlation between RT-qPCR experiments and RNA-seq data (R^2^ = 0.9379) (Figure S6). Five out of 20 selected DEGs were upregulated in the F-subpopulation (Ctr1 ID:1092022, EF-Tu, *Img2*, Psd8 ID: 1062065 and *Sc15* ID:1094726) (see Tables S5 and S7). The rest (*Aco1* ID:1075708, *Ada2*, *Adr1* ID:176657, *Aif*, *Aox*, *Cda1* ID:1078467, *Fzo1*, *Gap1* ID:1092209, *Pdr15* ID:1091717, *Prz1* ID:161685, *Rco*-3 ID:1094871, *Sod1* ID:1113505, *Tpo1* ID:1094703, *Trx1*, and *Uap1* ID:1107060) were upregulated in the S-subpopulation (see Tables S6 and S8). The gene IDs are those of the reference genome MkPC15 available at www.genome.jgi.doe.gov/PleosPC15_2/PleosPC15_2.home.html, 8 October 2021.

#### 3.4.2. Gene Expression Profiles Explain Correlations between Genotype and Phenotype in Monokaryons Belonging to F- and S-Subpopulations

To investigate the genetic bases of the differences in growth rate between the F and S monokaryons, we focused on the *Aox*, *Sod1*, *Cyc7,* and *Cox5B* genes because they are related with oxidative stress and hypoxia. The two parental alleles of the *Aox* gene showed a 99% of protein sequence identity, though they differed in their promoters (data not shown). No differences (100% of identity) were observed between the alleles of the *Sod1* gene. Both genes, *Aox* and *Sod1*, mapped to chromosome VII (Figure 2). The *Cyc7* and *Cox5B* genes mapped to chromosome VIII as a QTL related to the growth rate in monokaryons. No differences were found between the alleles of parental protoclones of both genes (100% identity, data not shown). The RNA-seq experiments revealed significant expression differences (detected by RPKMs, Table 1) in the four genes depending on the parental type allele they received. Other genes overexpressed in hypoxia conditions in slow-growing monokaryons of *P. ostreatus* were *Ole1*, *Erg11,* and *Fet3* (a ferroxidase ID: 1094975) (Table S6).

### 3.5. In Situ Detection of Superoxide Radicals and Hydrogen Peroxide in Monokaryons Suggest Oxidative Stress

Due to hypoxia genes appearing upregulated in the monokaryons of the S-subpopulation, we investigated ROS production in the F- and S-subpopulations. The occurrence of superoxide radicals (O_2_^−^) was confirmed by the emergence of an intense dark-blue colored water-insoluble precipitate ring (formazan) of variable width, mainly on the mycelia tips of the S-subpopulation. The exceptions were Mk61 (it must be noted that this monokaryon produces a mainly floating biomass in SmF), which harbored *Aox*, *Sod1,* and *Cyc7* inherited from MkPC9; and Mk75, which also ferried the MkPC9 allele type of the *Cox5B* gene (Table 1). The color pattern production indicated a high accumulation of O_2_^−^ as observed in Mk13 and Mk20 of the F-subpopulation, both bearing alleles of the MkPC9 type. Mycelial sectors were another distinctive feature in most slow-growing monokaryons and some fast-growing ones (Figure S7A).

H_2_O_2_ production detected that Mk06 and Mk13 of the F-subpopulation secreted a halo of H_2_O_2_ into the culture media (Figure S7B). In addition, Mk13 and Mk20 also showed a dark central area indicating an accumulation of H_2_O_2_ radicals. Intensely colored mycelia appeared in most of the slow-growing monokaryons due to the in situ activity of the Sod1 enzyme. The upregulation of the *Sod1* gene was previously detected by RNA-seq experiments (Table 1).

### 3.6. Assessment of Lipid Peroxidation by MDA Levels in Monokaryons of F- and S-Subpopulations

To get evidence of oxidative stress, we analyzed the lipid peroxidation of cell membranes as a biomarker in mycelia of the F- and S-subpopulations by measuring the MDA equivalents, and we found that the average MDA equivalents were 6.1 ± 1.4 and 3.7 ± 0.46 nmol/g FW for the F- and S-subpopulations (Figure S8). A significant decrease (*p*-value < 0.001) of MDA levels was detected in monokaryons of the S-subpopulation. Furthermore, in slow-growing monokaryons of *P. ostreatus*, the *Ole1* and a cytochrome b5 reductase (*Cyb5R*, ID: 1102329) genes involved in these processes were overexpressed (Table S6).

### 3.7. Monokaryons of the F-Subpopulation Are More Sensitive to Chitosan

**Figure 5:**
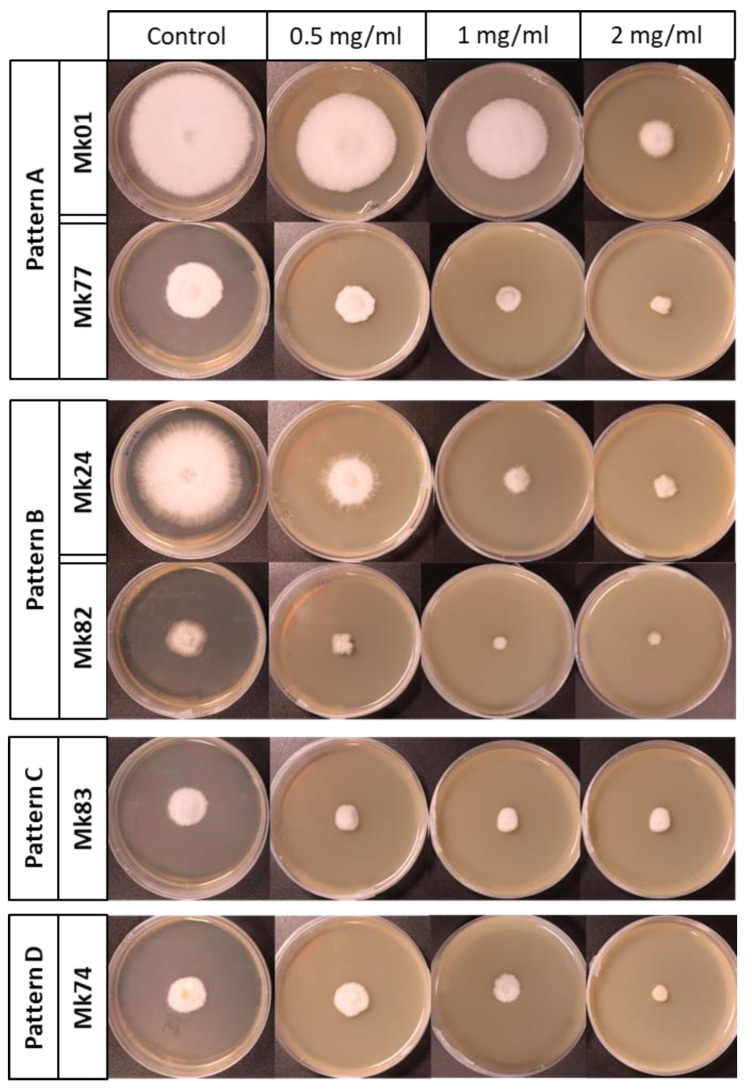
Growth patterns of monokaryons of the F- and S-subpopulations growing for 10 days on control medium (MESM) and MESM plus different doses of chitosan (0.5, 1, and 2 mg/mL).

We studied the effect of chitosan on the growth rate of 18 monokaryons grown on MESM amended with different chitosan concentrations. Analyzing the growth rate in the monokaryons of both subpopulations, we could establish four different patterns: A to D (Figure 5).

The monokaryons displaying the chitosan response pattern A in both subpopulations showed significant growth rate differences (*p*-value < 0.05) at all chitosan concentrations (Figure S9). The situation differed in monokaryons with pattern B, which showed no differences in the mycelial growth rate at 1 and 2 mg/mL chitosan, while significant differences (*p*-value < 0.05) were detected between the highest concentrations in comparison with the other ones. The monokaryons of both subpopulations displaying the pattern C showed no effect of chitosan on the growth rate at three chitosan concentrations, but differences were observed between them and the control sample. Only one monokaryon, Mk74, was assigned to pattern D. This monokaryon showed no reduction in growth rate when the control and the 0.5 mg/mL of chitosan samples were compared, but showed significant differences (*p*-value < 0.05) in comparison to the control at the highest chitosan concentrations (Figure S9).

The results obtained showed that patterns A (Mk01, Mk08, Mk13, Mk20, and Mk27) and B (Mk02, Mk06, Mk24, and Mk28) were predominant in the F-subpopulation, whereas monokaryons of the S-subpopulation were sorted in four different patterns: Pattern A (Mk62 and Mk77), B (Mk61, Mk75, Mk76, and Mk82), C (Mk69 and Mk83), and D (Mk74).

### 3.8. Effect of Chitosan Treatments on Some Genes Upregulated in Monokaryons of the F- and S-Subpopulations

RNA-seq experiments performed in this work showed that two chitin deacetylase *Cda1* genes were overexpressed in slow-growing monokaryons, suggesting they are chitosan producers. To determine how this production could affect growth rate and promote the expression of genes involved in oxidative stress and cellular detoxification, such as *Aox*, *Sod1,* and *Pdr15*, we analyzed the effect of different chitosan concentrations on the F- and S-subpopulations by assessing the expression profile of the genes mentioned above.

**Figure 6:**
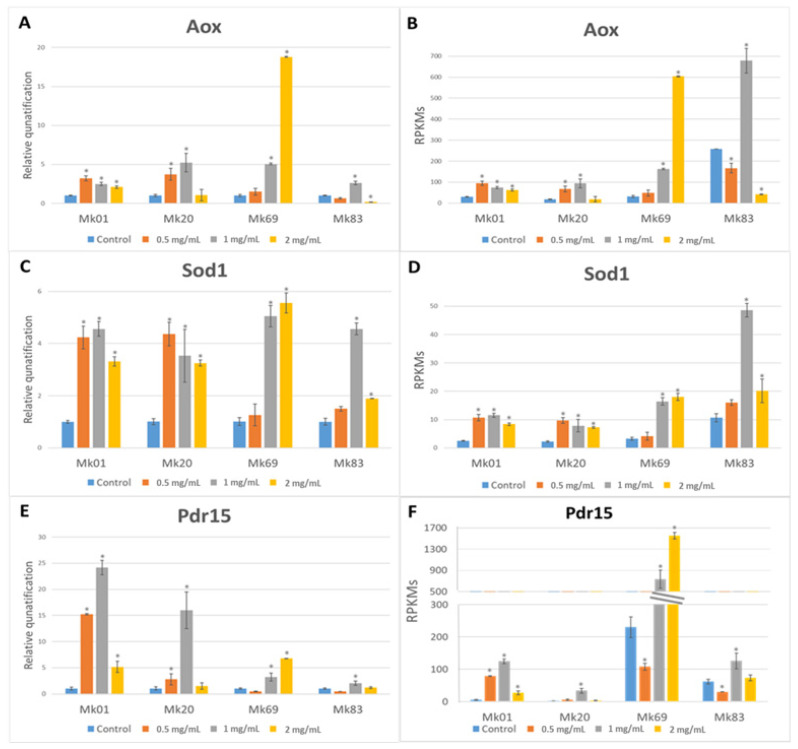
Effect of chitosan on *Aox*, *Sod1* and *Pdr15* expression. Relative quantification of the expression of (**A**) *Aox* gene, (**C**) *Sod1* gene, and (**E**) *Pdr15* gene in mycelia of fast (Mk01 and Mk20) and slow (Mk69 and Mk83) growing monokaryons grown on MESM containing different chitosan concentrations (0.5, 1, and 2 mg/mL). (**B**,**D**,**F**) estimate the RPKM values for *Aox*, *Sod1,* and *Pdr15* genes under different chitosan concentrations. Individual RPKMs were estimated using the RPKM values retrieved from the RNA-seq as the control multiplied by the relative quantification of the transcript obtained after RT-qPCR analysis of each monokaryon grown under different doses of chitosan. Asterisks indicate significant differences between control and chitosan doses (Tukey’s test, *p*-value < 0.05). That is, the relative expression of the *Aox* gene at 1 mg/mL in Mk69 was 5.0 fold the control. Assuming that the RPKM values obtained by RNA-seq were 32.1, the estimated RPKM values for the *Aox* gene would be 160.5.

The expression profile of the *Aox* gene was analyzed in two fast (Mk01 and Mk20) and two slow (MK69 and Mk83) growing monokaryons classified in the A and C patterns using RT-qPCR. The RPKM expression data were estimated as described in the Figure 6 legend.

Compared to the control sample, the fast-growing monokaryons increased their relative expression values, mainly at 0.5 and 1 mg/mL of chitosan. The highest (2 mg/mL) chitosan dose induced a limited expression increase in Mk01 but was ineffective in Mk20 (Figure 6A). A substantial growth rate reduction was observed upon chitosan addition (Figure S9) in both monokaryons. The relative expression of the *Aox* gene in slow-growing monokaryons revealed quite different landscapes. In Mk69, chitosan *Aox* induction was increased at high doses, while in Mk83, the highest response was obtained at 1 mg/mL (Figure 6A). It should be mentioned that the two slow-growing monokaryons showed a similar growth rate pattern (Figure S9) but different parental origins of the *Aox* alleles (Table 1). The estimated RPKMs of the *Aox* gene showed that the control sample of Mk83 displayed the highest value compared to the other control samples of the rest of the monokaryons (Figure 6B).

The expression analysis of the *Sod1* gene followed a relatively similar expression profile to the *Aox* gene (Figure 6C): the individuals with highest *Aox* expression had the highest *Sod1* RPKM values (Figure 6D). As both genes are placed at chromosome VII, and no crossover was detected, it could be said that both genes were inherited as a supergene.

The expression of the gene *Pdr15* was higher at lower chitosan concentrations, and at the highest concentration of this antifungal, the gene showed a mild effect on Mk01 and no effect on Mk20 (Figure 6E). The relative expression of *Pdr15* in slow-growing monokaryons displayed a similar profile to that observed in the *Sod1* gene. The estimation of RPKMs of the *Pdr15* gene (Figure 6F) was also analyzed. We observed that the control samples of the slow-growing monokaryons exhibited the highest RPKM values compared to the control samples of the fast-growing ones.

### 3.9. Analysis of Mitogen-Activated Protein Kinase (MAPK) Signalling Pathways in Monokaryons of the F- and S-Subpopulations

**Figure 7:**
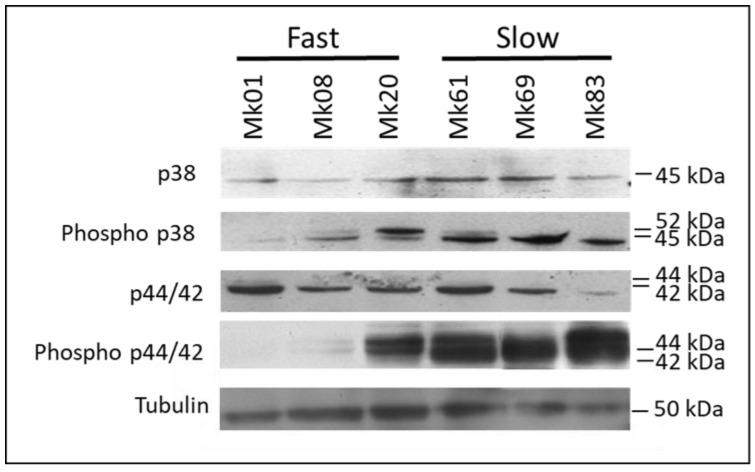
p38 and p44/42 MAPK in monokaryons of F- and S-subpopulations. Twenty five micrograms of total protein extract from three fast (Mk01, Mk08, and Mk20) and three slow (Mk61, Mk69, and Mk83) growing monokaryons cultivated in SMY liquid medium for seven days were subjected to immunoblot analysis with an anti-phospho-p38 MAPK antibody and an anti-phospho-p44/42 MAPK antibody to detect phosphorylation of these MAPKs. A monoclonal α-tubulin antibody was used as a loading control. The intensity of the phosphorylation signal is inversely correlated with the growth rate of mycelia.

We have studied the Mitogen-Activated Protein Kinase (MAPK) pathways in monokaryons of the F (Mk01, Mk08, and Mk20) and S (Mk61, Mk69, and Mk83) subpopulations. Total protein extracts of monokaryons were immunoblotted and revealed using commercial antibodies against the unphosphorylated and phosphorylated forms of p38 (HOG) and p44/42 (ERK1/2) of CWI pathways to measure the endogenous levels of both kinases in monokaryon protein extracts (Figure 7).

The unphosphorylated p38 (45 kDa) band showed a similar intensity in Mk20 (the slowest of the F-subpopulation) and the monokaryons of the S-subpopulation Mk61 and Mk69. When the phosphorylated version of p38 (Phospho p38), which recognizes the TGY motif characteristic of stress-activated MAPK activated by phosphorylation in threonine (T) and tyrosine (Y), was probed, two bands of 45 (phosphorylated p38) and 52 (unknown origin) kDa were detected. The 45 kDa band lighted up in two of the three fast-growing and the three slow-growing monokaryons.

We also analyzed the CWI pathway in fast- and slow-growing monokaryons. The antibodies against p44/42 detected a predominant band of 42 kDa with a similar intensity in all samples except in the fast-growing Mk83 that was fainter. In contrast, the antibody against phosphorylated p44/42 showed a signal in the slow-growing monokaryons and the fast-growing Mk20. We analyzed if any correlation existed between the strength intensity signal of the bands and RPKM values of the *Cda1* genes (data not shown). The results showed that the high activation level of the p44/42 protein corresponded with the highest RPKM values of the Cda1 genes of the slow-growing monokaryons, where these genes were overexpressed, and chitosan was produced.

## 4. Discussions

A global discussion of all of the topics developed in this paper is presented. Firstly, we focus on the mycelial growth rate and cell size in fast- and slow-growing monokaryotic subpopulation progenies obtained from the dikaryotic strain DkN001 in 2016, compared with the same data recorded from similar subpopulations obtained from the same dikaryon in 1998 [68]. In fact, we have seen that the progeny obtained in 2016 showed a drastic decrease in mycelia growth rate compared to that of 1998 [52]. In this sense, the microscope analysis of mycelial hyphae of F- and S-subpopulations described in this paper (Figure S2) showed that both had a similar diameter. However, increased septation was only present in hyphae of monokaryons belonging to the S-subpopulation. Chiu et al. [69] described that yeast cells subjected to some oxidative stresses delay entry at the G1 phase of the cell cycle, allowing them to repair any cellular damage. This observation means that oxidative stress sensing would be coordinated with the regulation of the cell cycle. The increase in the number of septa observed in monokaryons of the S-subpopulation is compatible with a curtailment of the cell cycle, probably due to endogenous changes, leading to metabolic switching.

Differences between the two subpopulations of monokaryons uncovered by the combined analysis of the whole-genome transcriptome and SNPs revealed: (i) that the different phenotypic features observed in monokaryons of both subpopulations were based on allele differences and (ii) that genetic recombination in *P. ostreatus* was restricted to telomeric and subtelomeric regions, as has been reported in other basidiomycetes [70]. However, in *Pleurotus*, a lack of CO in some chromosomes was observed. This last fact assures that a QTL for monokaryotic growth rate placed in chromosome VIII, apart from some other genes, was inherited as a supergene, and it was a clear-cut example of a low genome variability observed in the progeny of *P. ostreatus*.

Data analysis of the whole-genome transcriptome profile of both subpopulations yielded different landscapes with genes overexpressed in the S-subpopulation that inform about significant metabolic changes. Although the traditional loss-of-function analysis has been a straightforward mechanism to uncover a gene function, the overexpression analysis is an alternative tool to identify pathway components that may remain undetected using a knock-out approach [71], even more, when overexpression mirrored the physiology of the slow-growing monokaryons, as is shown in this paper,

We jointly analyzed DEGs (in this paper, overexpressed genes) and genes PC and NC with the growth rate in both subpopulations and found that the transcriptome profile yielded 900 genes correlated with growth rate and 531 DEGs (as was described in Section 3.4). About 73% of transcripts were genes PC with growth rate in the F-subpopulation, but only 38.4% were F-DEGs. Most of them belonged to cellular processing and signaling (KOGs: O, U, T, and Z) and information storage and processing (KOGs: J, A, and K) (Table S9), which indicated that monokaryons of the F-subpopulation showed signals of active growth under the experimental conditions described in this work. By contrast, monokaryons of the S-subpopulation showed only 10.2% of genes were NC with growth rate, and about 61.6% were S-DEGs. It should be noticed that 11.8% of S-DEGs were located in chromosome VIII, and most of them were related to the metabolism and transport of different molecules. Genes found in the S-subpopulation, mainly oxidative stress genes, undoubtedly indicated that other metabolic conditions, different from those found in fast-growing monokaryons, have been developed under identical culture conditions, which have led to symptoms of strain degeneration. Suparmin et al. [72] obtained similar results under identical experimental conditions, though the metabolic changes observed in the monokaryons of F- and S-subpopulations shed light on the different genetic backgrounds of both subpopulations. Overlapping analysis performed between F-DEGs and PC genes versus S-DEGs and NC genes using Gene Ontology (GO) (Figure S4) reinforced, for each subpopulation, the data obtained by KOG analysis.

A combined analysis of MkPC9 and MkPC15 SNPs and RNA-seq experiments carried out in the parental protoclones and in the progeny of the F- and S-subpopulations shed light on the performance of the monokaryons growth rate in liquid and solid media. This clue was provided by the analysis of RNA-seq experiments of four genes and growth rate QTLs. The genes analyzed were: (i) *Aox* coding for a non-energy-conserving terminal oxidase of the Electron Transport Chain (ETC) with a lower affinity for oxygen than cytochrome oxidase, and overexpressed under stress conditions [73,74]; (ii) *Sod1* encoding a superoxide dismutase which regulates the production of ROS and is required for tolerance to oxidative stress [75,76]; (iii) *Cyc7* (isoform 2 of cytochrome c, ID:1113744) that is an electron carrier protein predominant under anaerobic/hypoxic conditions; (iv) and *Cox5B* (cytochrome c oxidase subunit 5B, ID:1094413) that is a subunit of the ETC Complex IV expressed under hypoxic conditions [77].

We observed that expression levels of the *Aox* and *Sod1* genes in the progeny depended on the parental allele inherited. The high levels of the *Aox* gene in the S-subpopulation suggested an impairment or not a fully functional respiratory chain, which could increase intracellular ROS. These high ROS-induced *Aox* levels could be compensated with high induction levels of the *Sod1* gene, the overexpression of which decreased cellular susceptibility to oxidative stress by promoting cell adaptation as described by Li et al. [78] in cancer cells. Both genes, cosegregated in the progeny and their expression and the in situ ROS detection in monokaryons allowed us: (i) to differentiate both subpopulations, (ii) to corroborate the intense oxidative stress supported by monokaryons of the S-subpopulation, and (iii) to show the link between the genotype and the phenotype performance observed in the progeny. This link was also observed when the hypoxia-induced genes *Cyc7* and *Cox5B* were studied. Kwast et al. [77] observed that mitochondrial cytochrome c oxidase (COX) subunit 5 and cytochrome c (Cyc) existed in two isoforms in yeast, which were transcriptionally regulated by oxygen. The gene pair *Cox5A*/*Cyc1* encodes the normoxic isoforms (Cox5A and iso1-Cyc), while the gene pair *Cox5B*/*Cyc7* encodes the hypoxic isoforms (Cox5B and iso2-Cyc). Castello et al. [79] described that the hypoxic genes *Cyc7* and *Ole1* were induced at higher levels in yeast strains carrying the Cox5B isozyme. Rox1 is a transcriptional repressor of *Cox5B* and *Cyc7* genes in normoxia. In *P. ostreatus*, the expression of the *Rox1* homolog gene (ID: 1017219, Table S7) was positively correlated with growth rate, which means that its downregulation in monokaryons of the S-subpopulation led to high expression of the hypoxic isoforms Cox5B and Cyc7.

As no differences between the parental alleles (100% of protein sequence identity) of the loci *Cox5B*/*Cyc7* were found, the differences in the expression levels between fast- and slow-growing monokaryons could depend on *Rox1* gene expression. In fact, it could be accepted that the expression of Rox1-regulated hypoxic genes would increase when oxygen concentrations decrease. Liu et al. [80] showed that, in yeast, oxidative stress induced the expression of the OXPHOS-related *Cox5B* and *Cyc7* genes through a mechanism that released the transcriptional repressor Rox1 from their promoters. All these data show that monokaryons of the S-subpopulation showing strain degeneration are under intense oxidative stress.

The imbalance of ROS leads to an excess of radical species that produce adverse modifications to cell components such as lipids, proteins, and DNA damage [81]. As we detected by a proper staining method, the activity of the Sod1 enzyme, especially in slow-growing monokaryons, we assessed the malondialdehyde levels (MDA, a biomarker of oxidative stress depending on the composition of saturated/unsaturated fatty acids present in cell membranes, the substrate for free radicals) resulting from peroxidation of biological membranes in fast- and slow-growing monokaryons [67]. Lipid peroxidation is a free radical-mediated chain of reactions that, once initiated, results in an oxidative deterioration of polyunsaturated fatty acids (PUFA) [82]. High levels of MDA correspond to cell membranes containing a high percentage of PUFA, while low levels of MDA correspond to membranes with a high percentage of Saturated Fatty Acids (SFA).

Stearoyl-CoA desaturase (SCD) is an enzyme that catalyzes the conversion of SFA into their corresponding monounsaturated fatty acids (MUFA). In *P. ostreatus*, the *Ole1* homolog gene encodes an SCD enzyme, which is an endoplasmic reticulum resident enzyme that requires oxygen, NADP(H), and an electron transport chain that contains, among others, the FAD-dependent cytochrome b5 reductase, for which the primary substrates are palmitic (C16:0) and stearic acids (C18:0). The desaturation of these acyl-CoA fatty acids yields MUFAs such as palmitoleic (C16:1 n-7) and oleic acid (C18:1 n-9), respectively [83].

Due to their high PUFAs, cellular membranes or organelle membranes are especially susceptible to ROS damage producing lipid peroxidation. This process, in which free radical species (such as oxyl, peroxyl, and hydroxyl radicals) remove electrons from lipids, produce reactive intermediates that can undergo further reactions [84]. The lower MDA levels observed in the S-subpopulation monokaryons than those obtained in the F-subpopulation could suggest a higher membrane strength supported by the overexpression of the hypoxic *Cyc7*, *Cox5B,* and *Ole1* genes. The overexpression of the *Cyc7* and *Cox5B* genes would indicate that oxygen availability plays a crucial role in the composition of cell membranes.

The *Ole1* gene encodes a stearoyl ∆^9^ desaturase regulated at transcriptional (by Rox1 [77]) and post-transcriptional levels by different factors such as glucose, fructose, cholesterol, PUFAs, and senescence, among others. This enzyme catalyzes the conversion of SFA to MUFA. Different functions have been assigned to this enzyme. Still, the most important one is to maintain the fatty acid composition and membrane structure because high levels of SCD could lead to rigid membrane composition (low membrane fluidity), apoptosis resistance [83,85], and extension of lifespan as was observed in monokaryons of the S-subpopulation. Nasution et al. [86] described that *Ole1* overexpression might enhance tolerance to various types of stress because it would produce an accumulation of MUFA, which determines a lower membrane permeability. It was observed that cell membranes of some entomopathogenic fungi belonging to the *Cordycipitaceae* family and nematophagous egg-parasitic fungi of the *Clavicipitaceae* families changed their membrane fluidity to reduce chitosan damage [40].

Chitosan was described as a powerful biocidal [87], affecting the mycelial growth rate and the expression of genes related to cell detoxification and hypoxia. The effect of chitosan on growth rate was studied in monokaryons of the F- and S-subpopulations, and the results obtained indicated that the decrease in growth rate was genotype dependent. In this sense, most fast-growing monokaryons ferried the growth rate QTL of the MkPC9 type and the Cox5B and Cyc7 allele expression compatible with normoxia conditions. Conversely, slow-growing monokaryons harbored the growth rate QTL of the MkPC15 type, the *Cox5B* and *Cyc7* alleles expressed under hypoxia conditions, and displayed mycelial degeneration (Table 1 and Figure 2).

Palma-Guerrero et al. [40] related fungal chitosan sensitivity to high levels of PUFA and increased plasma membrane fluidity. In the light of that result, we speculated that monokaryons of the F-subpopulation could have a higher level of PUFAs than those of the S-subpopulation because their growth rate was reduced in the presence of chitosan, and their MDA levels were higher than those observed in slow-growing monokaryons overexpressing the *Ole1* gene.

Because chitosan induces membrane permeation and increases the production of intracellular ROS [28], the response of monokaryons of the F- and S-subpopulations to different chitosan concentrations was studied by analyzing the expression levels of the *Aox* and *Sod1* genes. The high expression of the *Aox* gene at 2 mg/mL of chitosan in Mk69 could be explained as an adaptive response of the monokaryotic mycelium grown for 10 days under different chitosan concentrations. Thus, these culture conditions would lead to a stimulation effect of the alternative respiration due to increasing *Aox* transcripts. By contrast, constitutive expression of *Aox* and *Sod1* genes in the control sample of Mk83, which inherited MkPC15 type alleles, could fit with a monokaryon already adapted to chitosan (resistant to chitosan). We hypothesized that *Aox* would sense the ROS production, and *Sod1* would stimulate a chitosan defense/adaptive reaction due to both genes showing an increase of their transcripts in those monokaryons with inherited MkPC9 type alleles. Knock-out experiments alternatively in both genes could provide some additional clues.

Many stress response genes were overexpressed in the whole transcriptome profile of monokaryons of the S-subpopulation. One of them was the *Ada2* gene, a histone acetyltransferase coactivator of the SAGA complex, which can bind to about 200 gene promoters including those related to glycolysis, pyruvate metabolism, oxidative stress, drug responses, cell wall response [88], and genes involved in ergosterol biosynthesis [89].

This paper focused on the *Pdr15* gene, which was described as the first pleiotropic drug-resistant ABC transporter involved in a general stress response [90] in monokaryons of the F- and S-subpopulations. The expression of this gene was increased in fast-growing monokaryons, probably due to the ROS insult produced by chitosan. The increase of relative expression of this detoxification gene could indicate how fast-growing monokaryons fight off different chitosan concentrations. By contrast, the behavior showed by the slow-growing monokaryons could be understood as the result of an adaptive response/resistance mechanism developed to cope with different chitosan concentrations. This finding could suggest that the *Pdr15* gene, as the other genes mentioned above, were constitutively expressed in the S-subpopulation to alleviate ROS and altogether could constitute markers of strain degenerated mycelia.

Due to the RT-qPCR experiments being carried out in the stationary phase (ten-day culture under chitosan treatment), we assumed that nutrient availability was insufficient, as shown by the overexpression of the hexose transporter Rco-3 (ID:1094871, Table S10). Thus, we supposed that toxic metabolites and catabolites had started to accumulate, yielding cellular damage and the overexpression of some apoptotic genes, such as an aspartic protease Ap1 (ID:1033895), an apoptosis-inducing factor Aif1 (ID:1113817), and a metacaspase Mca1 (ID:1114660) (Table S10); despite the overexpression of the *Ole1* gene, which is involved in the extension of lifespan [83]. Thus, in slow-growing monokaryons, we suggest that *Pdr15* could be induced to alleviate oxidative stress as described in yeast [90], and together with *Aox*, *Sod1,* and *Ole1* genes, could be used to distinguish mycelia of strain degenerated strains.

Other genes upregulated by the *Ada2* gene, the overexpression of which was independent of chitosan addition [88], were observed in slow-growing monokaryons. The list encompasses genes such as (i) *Erg1* (squalene synthase) and *Erg9* (squalene epoxidase) (Table S10), which are considered sterol precursors involved in the ergosterol biosynthesis pathway and their overexpression is the result of a depletion of cellular ergosterol in hypoxic conditions [91], (ii) the Prz1 transcription factor, a *Schizosaccharomyces* homolog to Crz1 [92], regulated by the Ca_2_^+^/calmodulin-dependent protein phosphatase (calcineurin) and activated under certain environmental conditions, and (iii) the *Pho84* gene, a pleiotropic high-affinity inorganic phosphate transporter the overexpression of which triggers the Endoplasmic Reticulum Unfolded Protein Response (ER-UPR) related to the extension of Replicative LifeSpan (RLS) in the retrograde response in yeast [12].

Changes in environmental conditions demand quick shifts in cell wall restructuring via the synthesis of chitin and chitosan enzymes. In this work, we have seen that monokaryons of the S-subpopulation overexpressed the *Uap1* and two *Cda1* genes, both involved in cell wall remodeling [93]. As previously described, the *Uap1* gene synthesizes chitin monomers, while Cda1 genes code for the enzymes necessary to deacetylate either the chitin polymer or the chitin monomer resulting from the activity of the *Uap1* gene with the aim of reshaping the cell wall. In *Cryptococcus* [94], the *Cda* genes are expressed during vegetative growth to provide integrity and proper rigidity to the cell wall and cope with the effect of self-chitosan production. Recently, Rizzi et al. [95] described *Cda* genes as necessary for cell wall integrity and viability.

It was described that the contact between *Trichoderma*, a mycoparasitic fungus, with its prey triggered a burst of oxidative stress detected by an increase of expression of chitin synthase and chitin deacetylase genes [96]. *P. ostreatus* is a basidiomycete with the predatory lifestyle of a nematode-trapping fungus. This lifestyle has evolved among cellulolytic or ligninolytic fungi due to nutrient deficiencies in nitrogen-limiting habitats [97]. We rule out that the increase of *Uap1* and *Cda1* genes were due to contact with any prey because experiments were performed under controlled conditions. Thus, we speculate that the overexpression of *Uap1* and *Cda1* genes in slow-growing monokaryons of *P. ostreatus*, with symptoms of strain degeneration, could be accounted for: (i) as a self-defense mechanism acting as a response to a signal molecule, chitosan, with the purpose to strengthen cellular membranes and restructure cell walls. Remodeling of cell walls could permit it to stand harsh oxidative stress conditions which could lead to an extension of lifespan and could be assumed due to the overexpression of the *Pho84* gene, and (ii) due to nutrient depletion that takes place during the stationary phase when experiments were performed, which would trigger the recycling of the cell wall monomers.

Alternatively, strain degeneration in slow-growing monokaryons could result from the impairment between nuclear and mitochondrial genes related to the functioning of the ETC. In *P. ostreatus*, mating between compatible mycelia entails the migration of nuclei (highly unusual mitochondrial migration). Hence, the newly formed dikaryon and its progeny only bring a mitochondrial type. This fact could be responsible for the weak performance of slow-growing monokaryons with a long-lasting cellular cohabitation period between unmatched nuclear and mitochondrial ETC genes. Currently, this fungal aspect in *P. ostreatus* is under study in our laboratory.

Taken together, we conclude that strain degeneration signs observed in slow-growing monokaryons of *P. ostreatus* could be the result of multiple factors that determine that strain degeneration can be seen as a pleiotropic trait.

Two pathways, the High Osmolarity Glycerol (HOG) and Cell Wall Integrity (CWI), regulate responses to stress conditions [98]. The HOG pathway is activated by different stresses [99,100], while the CWI pathway is necessary for the integrity of the cell wall [101]. The first one, once activated, coordinates the hyperosmotic stress response, though it could also be activated in response to other stresses, including oxidative [102], acid [103], methylglyoxal [104], temperature downshift [105], or heat stresses [106]. The CWI pathway is mainly activated by cell wall stress to ensure cell wall integrity by increasing chitin synthesis [96]. However, this pathway can be activated by some other stressful conditions, including hypo-osmotic shock [107], pheromone treatment [108], actin depolarization [109], the Unfolded Protein Response (UPR) [110], and oxidative stress, suggesting that although the pathway specificity is necessary, the HOG and CWI pathways can also be positively coordinated [98].

Immunoblots performed in mycelia of slow-growing monokaryons in the absence of chitosan showed the activation of the HOG pathway. Due to slow-growing monokaryons being themselves chitosan producers, it could be suggested that HOG activation is required to counteract the membrane insult resulting from this production in the slow-growing monokaryons and two of the three fast-growing monokaryons. Crosstalk between the stress response and the pleiotropic drug resistance *Pdr15p* was observed by Wolfgert et al. [90] because its induction bypasses upstream components of the HOG pathway. The overexpression of up to two *Pdr15* genes in slow-growing monokaryons (Table S10) revealed by RNA-seq experiments could suggest that its overexpression could also help HOG pathway activation.

Chitin synthesis and chitosan conversion are strongly influenced by external parameters, such as environmental stress factors. Perturbation of fungal cell wall synthesis triggers a compensatory response to ensure CWI by increasing chitin synthesis [96]. We assume that a perturbation of the fungal cell wall occurring in the slow-growing monokaryons and the fast-growing Mk20 accounted for the constitutive overexpression of the *Uap1* gene responsible for the synthesis of chitin monomers. This new synthesis, which would indicate cell wall restructuring, could activate the CWI pathway.

In summary, the results obtained provide evidence that (i) strain degeneration in *P. ostreatus* is linked to oxidative stress, (ii) the oxidative stress response in monokaryons of the F- and S-subpopulations is genotype dependent, (iii) marker genes of oxidative stress and cellular detoxification are highly expressed in monokaryons of the S-subpopulation with symptoms of strain degeneration, (iv) chitosan addition to monokaryons of both subpopulations uncovered the constitutive expression of *Aox*, *Sod1* and *Pdr15* genes in slow-growing monokaryons strains suggesting their adaptation to oxidative stress, and (v) the overexpression of the cell wall genes, *Uap1* and *Cda1*, in slow-growing monokaryons with a strain degeneration phenotype indicated cell wall reshaping and the activation of HOG and CWI pathways. Thus, these results could constitute a hallmark for mushroom producers to distinguish strain degeneration in commercial mushrooms.

## Figures and Tables

**Table 1:** Expression profile (RPKM) and parental origin allele of *Sod1*, *Aox*, *Cyc7*, and *Cox5B* genes. RPKMs were retrieved from RNA-seq data. MkPC9 (dark red) and MkPC15 (dark blue) are the parental protoclones. Light red (MkPC9 type) and blue (MkPC15 type) colors in fast- and slow-growing monokaryons indicate the origin of the parental allele in each monokaryon. Monokaryon growth rates ranged from the fastest to the slowest (indicated by the symbols + and -, respectively).

**Fast Growing Monokaryons**												
**Chromosome**	**Genes ID**	**Name**	**PC9**	**Mk01**	**Mk08**	**Mk20**	**Mk28**	**Mk13**	**Mk27**	**Mk24**	**Mk02**	**Mk06**
VII	1113505	Sod1	1.72	2.52	0.76	2.22	1.33	1.28	35.37	6.53	1.50	0.46
VII	1106868	Aox	26.08	29.59	24.70	17.99	21.08	22.96	59.23	136.92	31.39	12.44
VIII	1113744	Cyc7	580.99	1013.51	718.25	309.61	595.12	531.36	352.51	973.96	420.19	287.56
VIII	1094413	Cox5B	673.80	482.00	528.72	545.66	472.96	514.22	241.54	517.18	415.76	494.22
**Slow Growing Monokaryons**												
**Chromosome**	**Genes ID**	**Name**	**Mk74**	**Mk61**	**Mk75**	**Mk76**	**Mk77**	**Mk69**	**Mk82**	**Mk62**	**Mk83**	**PC15**
VII	1113505	Sod1	12.67	4.60	28.01	17.08	22.29	3.25	6.71	24.64	10.65	15.35
VII	1106868	Aox	176.59	46.42	254.42	252.21	274.65	32.13	276.74	187.39	258.25	168.44
VIII	1113744	Cyc7	1128.50	567.12	1427.61	806.00	846.54	770.25	1202.51	555.19	1180.23	1263.15
VIII	1094413	Cox5B	894.18	688.43	579.39	755.75	780.44	700.26	626.91	722.86	499.08	695.52
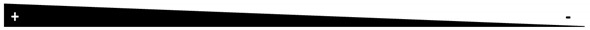												

## Data Availability

The data presented in this study are available on request from the corresponding author. The data are not publicly available because other data from these whole-genome transcriptomes are being used for other analyses to be published independently of this one.

## Supplementary Materials

The following are available online at https://www.mdpi.com/article/10.3390/jof7100862/s1: Supplementary Figure S1: Growth rate of the 60 monokaryons progeny. Supplementary Figure S2: Distances between septa of the F- and S-subpopulations. Supplementary Figure S3: VCF file related to SNPs. Supplementary Figure S4: Venn diagrams showing common and unique DEGs and genes correlated with growth rate in F- and S-subpopulations. Supplementary Figure S5: GO terms of overlapping genes in F- and S-subpopulations. Supplementary Figure S6: Validation of RNA-seq data by RT-qPCR assay. Supplementary Figure S7: In situ detection of ROS. Supplementary Figure S8: Analysis of lipid peroxidation. Supplementary Figure S9: Chitosan effect on *P. ostreatus* growth rate. Supplementary Table S1: List of primers. Supplementary Table S2: Clustering. Supplementary Table S3: Analysis of crossovers. Supplementary Table S4: Number of COs. Supplementary Table S5: DEGs in F-subpopulation. Supplementary Table S6: DEGs in S-subpopulation. Supplementary Table S7: Genes positively correlated with growth rate. Supplementary Table S8: Genes negatively correlated with growth rate. Supplementary Table S9: Number of genes in each KOG category. Supplementary Table S10: DEGs and correlated genes with growth rate related to different cell pathways.
