# Supplementary material for: Strain Degeneration in Pleurotus ostreatus: A Genotype Dependent Oxidative Stress Process Which Triggers Oxidative Stress, Cellular Detoxifying and Cell Wall Reshaping Genes
Source: J Fungi (Basel). 2021 Oct 14;7(10):862. doi: 10.3390/jof7100862 (PMC8537115; doi:10.3390/jof7100862)
Supplement: Supplementary file 1 [file jof-07-00862-s001.zip › jof-1400654 supplementary file/Supplementary figures.pdf]

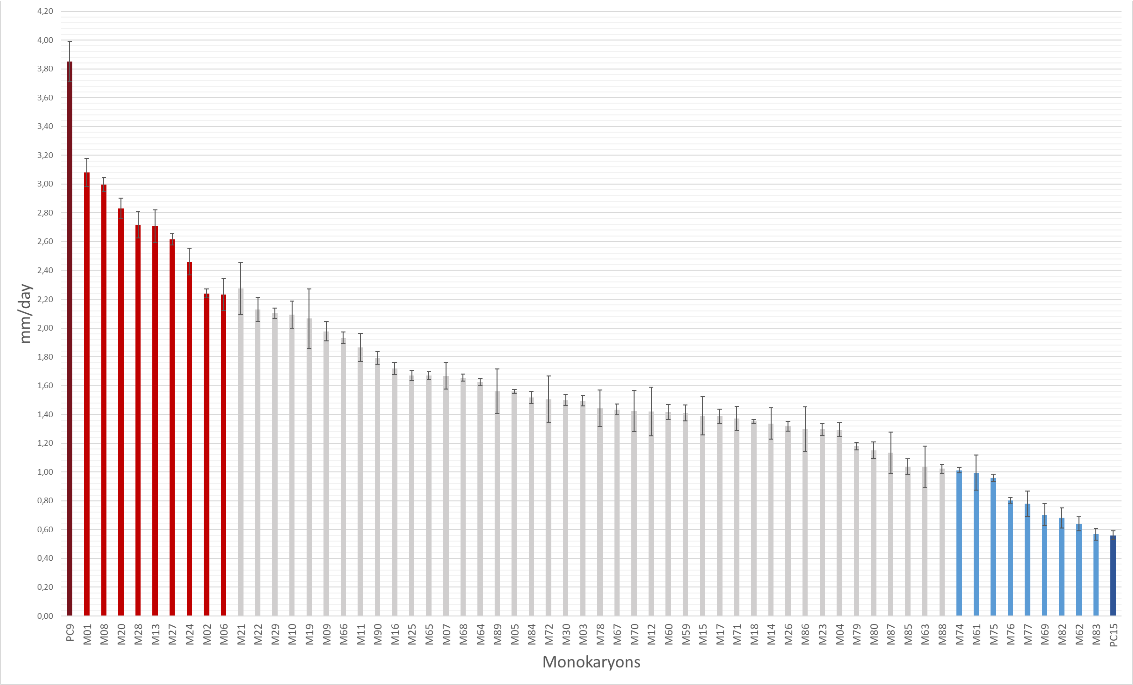

Figure S1: Growth rate of the 60 monokaryons progeny obtained from the *Pleurotus ostreatus* dikaryotic strain DkN001. Nine fast (red) and nine slow-growing monokaryons (blue), as well as the monokaryotic protoclons MkPC9 (dark red) and MkPC15 (dark blue) obtained after the de-dikaryotization of DkN001.

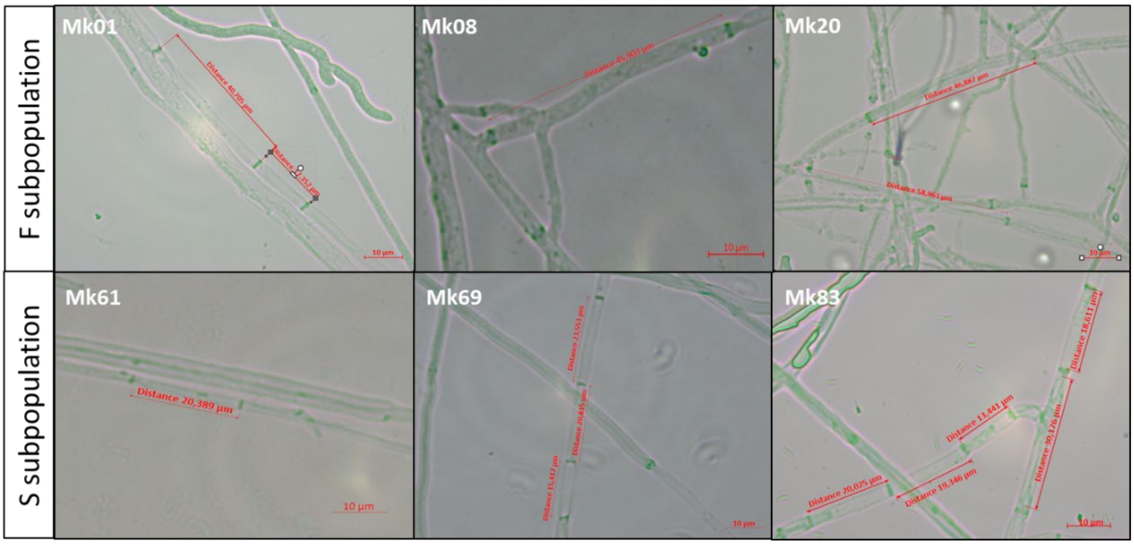

Figure S2: Distances between septa of monokaryons of the F and S-subpopulations. This figure shows a light microscope images of monokaryons of F (Mks01, 08 and 20) and S (Mks 61, 69 and 83) subpopulations obtained at 100X. Their distances between septa were analyzed using ZEN 3.0 software (Carl Zeiss Microscopy GmbH) and they are displayed in color red with a scale bar of 10 μm.

A

##fileformat=VCFv4.2
##reference=PC15v2 & PC9v1

#CHROM	POS	ID	REF	ALT	QUAL	FILTER	INFO
1	6468	171890-1	T	C	.	PASS	.
1	6658	171890-2	T	C	.	PASS	.
1	6740	171890-3	G	T	.	PASS	.
1	28156	153847-1	C	T	.	PASS	.
1	28173	153847-2	G	C	.	PASS	.
1	28185	153847-3	G	T	.	PASS	.
1	28203	153847-4	A	G	.	PASS	.
1	28205	153847-5	G	C	.	PASS	.
1	28209	153847-6	C	T	.	PASS	.
1	28231	153847-7	T	C	.	PASS	.
1	28279	153847-8	A	G	.	PASS	.
1	28317	153847-9	C	T	.	PASS	.
1	28334	153847-10	T	C	.	PASS	.
1	28342	153847-11	G	A	.	PASS	.
1	28357	153847-12	G	A	.	PASS	.
1	28365	153847-13	T	C	.	PASS	.

B

contig	position	variantID	PC15	PC9	PC15-Counts	PC9-Counts
scaffold_01	1912987	1032808-25	G	A	0	17
scaffold_01	1913357	1032808-26	A	G	0	9
scaffold_01	1913544	1032808-28	C	G	0	15
scaffold_01	1913722	1032808-29	A	G	0	8
scaffold_01	1913986	1032808-30	A	G	0	13
scaffold_01	1914220	1032808-31	G	A	0	3
scaffold_01	1914282	1032808-32	A	G	0	1
scaffold_01	1914443	1032808-33	C	T	0	4
scaffold_01	1924113	1031151-1	T	G	186	1
scaffold_01	1924195	1031151-2	T	A	280	1
scaffold_01	1924249	1031151-3	A	G	343	0
scaffold_01	1924252	1031151-4	C	T	343	0
scaffold_01	1924330	1031151-5	T	C	288	0
scaffold_01	1924361	1031151-6	C	T	130	0
scaffold_01	1924363	1031151-7	T	G	124	0
scaffold_01	1924365	1031151-8	T	A	123	0
scaffold_01	1924374	1031151-9	A	G	123	0
scaffold_01	1924452	1031151-10	G	A	259	0

Figure S3. A) VCF file containing information related to SNPs. 190,490 SNPs appeared between MkPC9 and MkPC15. CHROM: Chromosome number; POS: position in the chromosome; ID: gene ID in the MkPC15 genome reference (Joint Genome Institute, JGI), REF: nucleotide in the reference genome (MkPC15), ALT: alternative nucleotide in the MkPC9 genome (SNP). **B**) Example of the location (box) in the nucleotide 1,924,113 of scaffold_01 of a crossover in monokaryon 01 (Mk01).

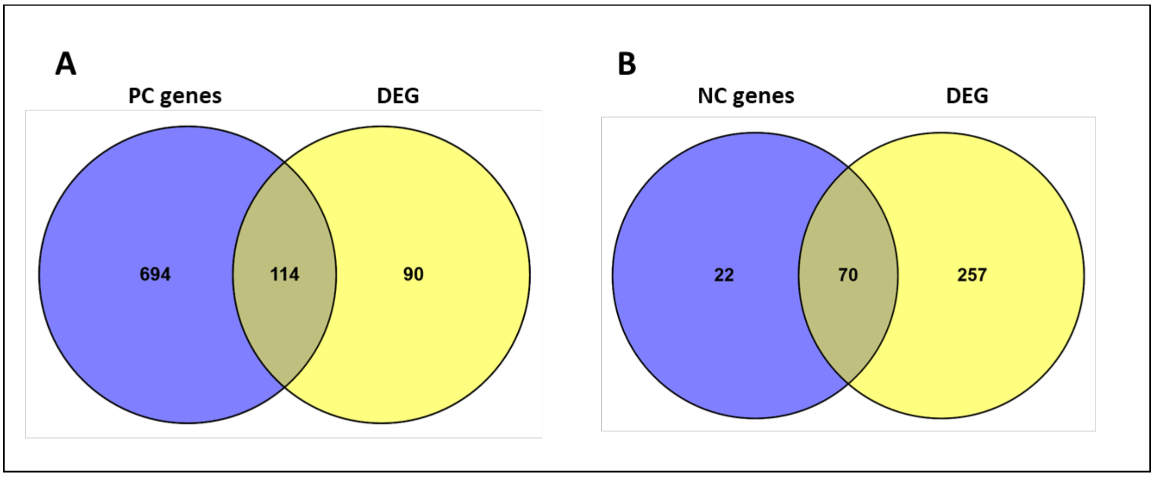

Figure S4: Venn diagram showing common and unique DEGs and genes correlated with growth rate in the fast (A) and slow (B) subpopulations.

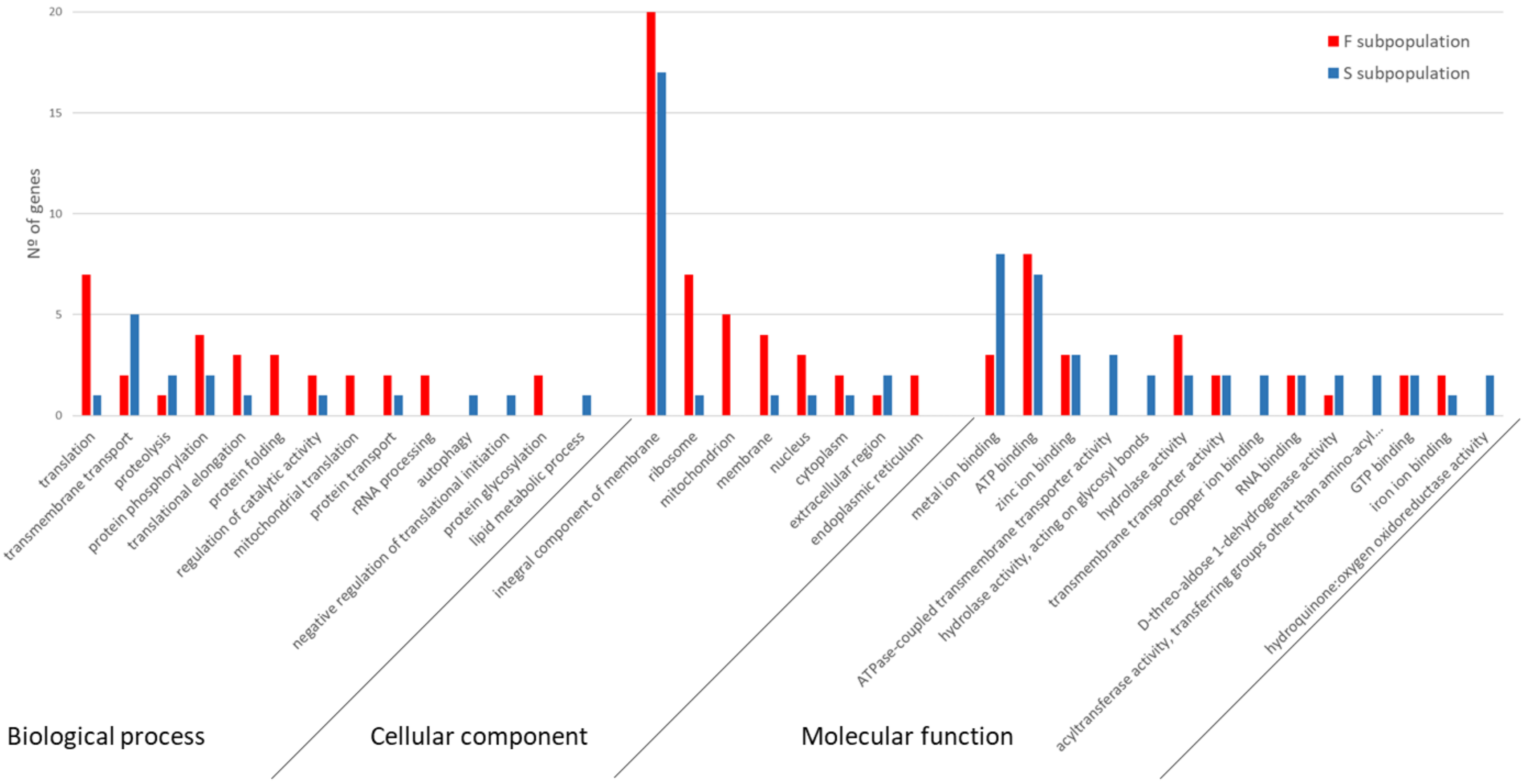

Figure S5: GO terms of overlapping genes in F and S-subpopulations. The X-axis shows the different functions assigned to each GO term found in the overlapping genes (F-DEGs/PC genes; S-DEGs/NC genes) in each subpopulation. The Y-axis indicates the number of genes belonging to each GO term.

Figure S6. Validation of RNA-Seq data by RT-qPCR assay. 20 DEGs belonging to different functional KOG categories were selected to confirm RNA-seq data. The X-Y plot was generated from the ratio of fold change obtained by RNA-seq data and RT-qPCR A) Table with the name, identification number in the MkPC15 genome, fold change from RNA-seq *versus* RT-qPCR analysis, description, and KOG categories of each DEG validated. B) Plot of simple linear regression and the R^2 between fold change obtained by RNA-seq data (X) and fold change values (Y) obtained by RT-qPCR

A)

Gene name	Gene_id	FC (RNA-seq)	FC (RT-qPCR)	Description	KOG categories
Aco1[a]	1075708	1,84	1,71	Aconitase (EC 4.2.1.3)	C
Ada2[a]	1112521	1,84	1,66	Histone acetyltransferase of SAGA complex	K
Adr1[a]	176657	2,46	1,97	Putative transcriptional regulator	S
Aif[a]	1113817	2,72	2,67	Apoptosis-inducing factor	S
Aox[a]	1106868	5,99	4,94	Alternative oxidase	Q
Cda1[a]	1078467	4,54	3,97	Chitin deacetylase (EC 3.5.1.41)	O
Ctr1[b]	1092022	6,58	8,80	High affinity copper transporter (1.A.56.2.2)	S
EF-Tu[b]	1092267	2,2	2,84	Translation-elongation factor Tu	J
Fzo1[a]	1058073	2,12	1,81	Transmembrane GTPase-mitofusin (1.N.6.1.1)	O
Gap1[a]	1092209	1,99	1,72	Aminoacid permease	E
Img2[b]	1031833	2,03	2,62	54S ribosomal protein, mitochondrial	J
Pdr15[a]	1091717	21,59	19,92	Pleiotropic drug resistance (3.A.1.205.6)	Q
Prz1[a]	161685	26,48	25,40	Calcineurin responsive transcription factor	S
Psd8[b]	1062065	4,48	6,22	Phosphatidylserine decarboxylase	I
Rco-3[a]	1094871	4,31	3,63	Hexose transporter (2.A.1.1.39)	P
Sc15[b]	1094726	10,16	13,94	Secreted protein 15	S
Sod1[a]	1113505	3,61	2,50	Cu/Zn superoxide dismutase (EC 1.15.1.1)	P
Tpo1[a]	1094703	5,91	4,92	MFS polyamine transporter (2.A.1.2.16)	U
Trx1[a]	1097745	1,66	1,45	Thioredoxin-like protein	Q
Uap1[a]	1107060	5,88	10,58	UDP-N-acetylglucosamine diphosphorylase (EC 2.7.7.23)	M

[a] DEGs up regulated in S subpopulation

[b] DEGs up regulated in F subpopulation

B)

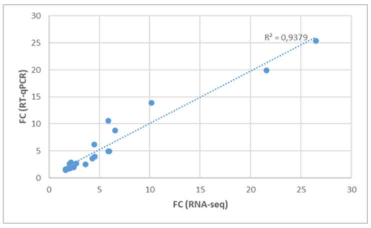

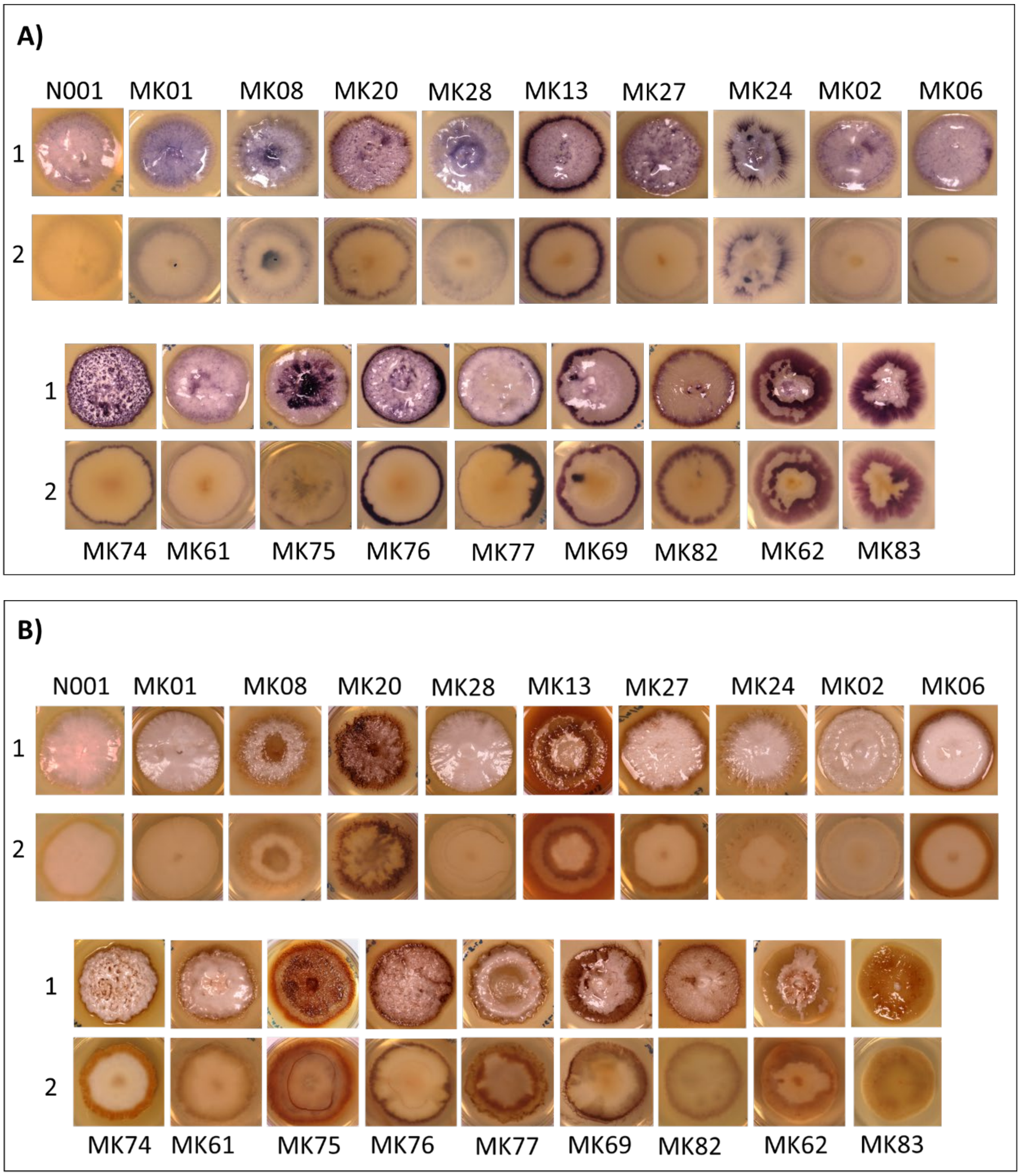

Figure S7. *In situ* detection of Reactive Oxygen Species (ROS) in mycelia (of about 30 ± 3 mm diameter) of the dikaryotic strain (DkN001) and monokaryons of the F (Mk01, Mk02, Mk06, Mk08, Mk13, Mk20, Mk24, Mk27 and Mk28) and S-subpopulations (Mk61, Mk62, Mk69, Mk74, Mk75, Mk76, Mk77, Mk82 and Mk83). A) Nitroblue tetrazolium (NBT) staining was used to detect superoxide radical (O_2^-) and B) Diaminobenzidine (DAB) staining for hydrogen peroxide (H_2O_2). Rows 1 and 2 correspond to the top and bottom of the Petri dish after staining. From left to right, monokaryons are arranged according to their decreasing growth rate**.**

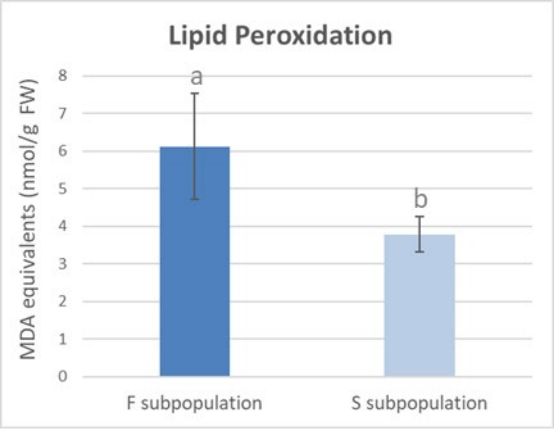

Figure S8. Analysis of lipid peroxidation in F and S subpopulations. The Y-axis indicates the MDA equivalents (nmol/g Fresh Weight). The X-axis shows the data average (n=9) of fast (dark blue) and slow (light blue) growing monokaryons of both subpopulations grown in SMY liquid medium (SmF) for seven days. Different lower case letters indicate significant differences of MDA equivalents between F and S-subpopulations (Student's test, p-value < 0.001).

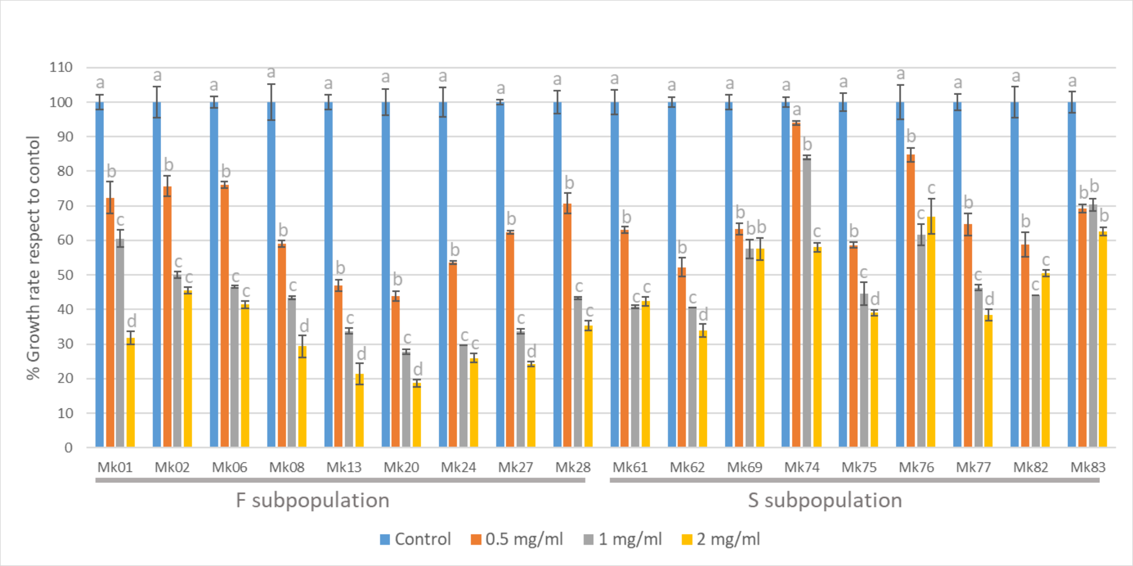

Figure S9. Chitosan effect on *P. ostreatus* growth rate. Monokaryons of F and S-subpopulations were grown on the control medium (MESM) and MESM plus different chitosan concentrations. Chitosan treatments and control were performed by triplicate. Bars show the data average (n=3) and SD (standard deviation). Lower case letters (a, b, c, d) indicate significant differences at level of *p*-value < 0.05 according to Scheffe's test (see Materials and Methods). For example, the Mk83 showed a mean decrease in the growth rate of 32.6 % when all chitosan doses were compared with control.
